# Maternal fasting during early gestation induces epigenetic alterations and schizophrenia-related phenotypes

**DOI:** 10.1038/s41380-026-03629-w

**Published:** 2026-05-13

**Authors:** Hongbo Wang, Miki Bundo, Yutaka Nakachi, Akinori Kanai, Yui Yamamoto, Hirofumi Miyazaki, Fumiko Toyoshima, Yasuyuki Shima, Mai Sakai, Zhiqian Yu, Hiroaki Tomita, Yutaka Suzuki, Kazuya Iwamoto, Yuji Owada, Motoko Maekawa

**Affiliations:** 1 Department of Organ Anatomy, Tohoku University Graduate School of Medicine, Sendai, Japan; 2 Department of Molecular Brain Science, Graduate School of Medical Sciences, Kumamoto University, Kumamoto, Japan; 3 Life Science Data Research Center, Graduate School of Frontier Sciences, The University of Tokyo, Kashiwa, Japan; 4 Department of Homeostatic Medicine, Medical Research Laboratory, Institute of Science Tokyo, Tokyo, Japan; 5 Neurodegenerative Disorders Collaborative Laboratory, RIKEN Center for Brain Science, Saitama, Japan; 6 Department of Psychiatric Nursing, Graduate School of Medicine, Tohoku University, Sendai, Japan; 7 Department of Psychiatry, Graduate School of Medicine, Tohoku University, Sendai, Japan; 8 Tohoku Medical Megabank Organization, Tohoku University, Sendai, Japan

**Keywords:** Schizophrenia, Neuroscience

## Abstract

Schizophrenia is a severe neurodevelopmental disorder whose etiology remains incompletely understood. Epidemiological studies of the Dutch Hunger Winter demonstrated that maternal famine during early gestation increased the risk of schizophrenia in offspring, implicating the Developmental Origins of Health and Disease (DOHaD) framework. However, the molecular mechanisms underlying this association remain unclear. Here, we developed a novel DOHaD-based schizophrenia model by subjecting pregnant mice to transient fasting restricted to the peri-implantation period, a critical window of global epigenomic reprogramming. Male offspring of fasted dams exhibited schizophrenia-related phenotypes, including impaired sensorimotor gating, abnormal behavioral patterns, and reduced dendritic spine density in the medial prefrontal cortex. Multi-omics profiling, integrating bulk RNA sequencing, Visium HD spatial transcriptomics, and DNA methylation arrays, revealed convergent alterations in synaptic organization, protein homeostasis, and oxidative stress pathways. These findings highlight how brief maternal fasting reprograms the epigenome and reshapes neural circuitry. Our work establishes the first animal model that directly mirrors early gestational famine exposure linked to schizophrenia risk, providing a unique platform for uncovering epigenetic mechanisms underlying the developmental origins of psychiatric disorders.

## Introduction

Schizophrenia is a severe, chronic neurodevelopmental disorder characterized by positive symptoms (hallucinations, delusions), negative symptoms (avolition, anhedonia, affective flattening), and cognitive deficits. Its lifetime prevalence is approximately 1% worldwide [1]. One of the key pathological findings is the reduction of dendritic spines in pyramidal neurons of the prefrontal cortex, which is linked to symptoms such as impaired sensorimotor gating [2–4]. Despite extensive research, the biological mechanisms driving schizophrenia remain unclear.

Epidemiological evidence has firmly implicated prenatal malnutrition in schizophrenia risk. In the Dutch famine of World War II, many pregnant women were exposed to acute starvation. Cohort studies revealed that male offspring of mothers exposed during the first trimester had nearly a twofold increased risk of schizophrenia [5–9]. This tragic “natural experiment” provided strong support for the Developmental Origins of Health and Disease (DOHaD) hypothesis, which posits that early-life adversity predisposes to adult disease through epigenetic programming [10–12].

DNA methylation is the most well-studied mechanism linking prenatal nutrition to long-term disease risk. Early embryogenesis is marked by global waves of DNA demethylation and de novo methylation [13–15]. Nutritional stress during this critical period may disrupt one-carbon metabolism (involving folate, methionine, and B vitamins). This disruption can reduce the availability of S-adenosylmethionine (SAM), the universal methyl donor [16]. Such limitation of methyl donor supply can interfere with the proper establishment of DNA methylation patterns [17, 18]. These alterations can persist across the lifespan and even across generations, reshaping gene expression without altering the DNA sequence. Indeed, genome-wide studies of individuals prenatally exposed to famine have identified differential DNA methylation, particularly among those exposed in the periconceptional period [19–21].

Rodent models have been widely used to explore the consequences of maternal malnutrition. Most studies have reported metabolic phenotypes in offspring following prenatal dietary restriction [22–26]. However, our previous study demonstrated that maternal lipid restriction induces schizophrenia-related outcomes, including behavioral abnormalities and altered DNA methylation [27]. These findings suggest that maternal nutrition impacts not only metabolic health but also neurodevelopment and psychiatric vulnerability.

Here, we developed a mouse model of transient maternal fasting confined to early gestation, designed to target an early gestational window analogous to early-trimester exposure to the Dutch Hunger Winter, and investigated whether such early nutritional stress induces persistent epigenetic remodeling and schizophrenia-related phenotypes in adult offspring.

## Materials and methods

### Mouse and housing conditions

Eight-week-old female and 10-week-old male C57BL/6 J mice were purchased from the Jackson Laboratory (Kanagawa, Japan) and housed in group of 3-4 per cage under a 12-h light/dark cycle (lights on at 08:00) with *ad libitum* access to a standard chow diet (MF diet; Oriental Yeast Co. Ltd., Tokyo, Japan) and water. All experimental procedures were approved by the Center for Laboratory Animal Research, Tohoku University (approval no. 2021 ido-067).

### Maternal fasting paradigm and breeding procedure

Mating was initiated by housing one male with 2–3 females overnight, with the presence of a vaginal plug defined as embryonic day 0.5 (E0.5). Pregnant females were randomly assigned to either the *ad libitum* control group (AL; hereafter referred to as AL) or fasting group (FAST; hereafter to as FAST).

Maternal fasting was applied at E1, E3, and E5 using an alternate-day fasting design, spanning the critical window encompassing global DNA demethylation and the subsequent re-establishment of the somatic DNA methylation signature [28, 29]. Given that mice consume two-thirds of their total food intake during the dark phase [30], a 16-h overnight fasting paradigm was chosen to induce a robust maternal fasting state. Moreover, between each fasting day, an *ad libitum* recovery phase was incorporated to allow us to effectively induce prenatal stress while minimizing the potential risk of pregnancy loss.

In the FAST group, food was removed from 17:00 to 9:00 on E1, E3, and E5. From E14 until parturition, pregnant females were housed individually. Pups were weaned on postnatal day 21 (P21). Offspring were used for behavioral testing and tissue collection at 10-13 weeks of age, with at least two litters represented per group to minimize litter effects. The number of litters used in major analyses is summarized in Suppl. Table 1.

### Golgi staining and dendritic spine analysis

Mice were anesthetized using inhaled isoflurane (FUJIFILM Wako Pure Chemical Corporation, Osaka, Japan). Briefly, mice were placed in an anesthesia chamber containing a paper pad soaked with isoflurane until deep anesthesia was achieved. During the procedure, anesthesia was maintained using a nose cone, and mice were transcardially perfused with the fixative provided in the SliceGolgi Kit together with its accessory reagent (Bioenno Tech, Santa Ana, CA, USA). Golgi staining was performed using the SliceGolgi Kit (Bioenno Tech, USA) according to the manufacturer’s protocol. Coronal sections (100 μm) of the medial prefrontal cortex (mPFC) were prepared and imaged by bright-field microscopy. Spine density was quantified on secondary apical dendrites longer than 70 μm, excluding the proximal 50 μm of the primary trunk, by an observer blinded to group assignments [31, 32] (see Supplementary Information for details).

### Behavioral tests

Prepulse inhibition (PPI), open field, and elevated plus maze (EPM) tests were performed in adult offspring using standard protocols, as described in the Supplementary Information. Briefly, PPI was assessed with SR-LAB startle systems, while locomotor activity and anxiety-like behavior were evaluated in the open field and EPM.

### Bulk RNA-seq analysis

Prefrontal cortices (PFCs) were collected from 10-week-old FAST and AL mice (n = 6 per group). Total RNA was extracted, and sequencing libraries were generated using the SMART method. After quality control, raw count matrices were analyzed in R (v4.4.2). Genes with fewer than 10 counts across samples were excluded. Differential expression analysis was performed using DESeq2 [33], and gene set enrichment analysis (GSEA) was conducted on ranked results with the clusterProfiler package [34].

Detailed procedures for library construction are provided in the Supplementary Information.

### Weighted gene co-expression network analysis (WGCNA)

For WGCNA, bulk RNA-seq data from both male and female offspring (10 weeks old, n = 6 per group per sex) were used. Gene expression counts were variance-stabilizing transformed (VST) using the DESeq2 package. Sex effects were removed using the removeBatchEffect function in the limma package [35]. A signed weighted gene co-expression network was constructed with the WGCNA package [36]. The soft-thresholding power (β = 9) was selected as the lowest power at which the scale-free topology fit index exceeded 0.85. The blockwiseModules function (minModuleSize = 50, mergeCutHeight = 0.35, and deepSplit = 2) was used for automatic network construction and module detection. In total, 14,871 genes were retained for network construction, of which 6270 genes were assigned to 31 co-expression modules. Module eigengenes were correlated with maternal fasting status using Pearson’s correlation coefficient, separately in male and female offspring. Hub gene networks were visualized using a modified version of the ModuleNetworkPlot function from the hdWGCNA package [37].

### Visium HD ST data analysis

Spatial transcriptomic analysis was performed on 10-week-old FAST and AL mice (n = 2 per group). Coronal PFC sections were processed with the Visium HD pipeline, and ~355,000 spots were retained after quality control (removal of low-count or high-mitochondrial spots, and genes detected in < 50 spots). Data were log1p-normalized and scaled to 10,000 per spot using Seurat. The medial prefrontal cortex (mPFC) region was manually delineated, and single-cell based spatial deconvolution was performed using the RCTD package [38]. Differential expression analysis was conducted using DESeq2 or the Seurat FindMarkers function (MAST method). Gene set enrichment analysis (GSEA) and over-representation analysis (ORA) were performed with clusterProfiler package [34]. Details of tissue processing and library construction are provided in the Supplementary Information.

### 10X FLEX single-cell data analysis

10X FLEX single-cell RNA-seq was performed on 10-week-old FAST and AL mice (n = 4 per group). For each group, pooled PFC tissue was fixed with formaldehyde and dissociated prior to library preparation. Details of preparation and quality control are described in the Supplementary Information. After QC, 13,696 cells (7,812 AL; 5,884 FAST) were retained. Data were log1p-normalized and scaled to 10,000 per cell in Seurat v5.3 (R v4.4.2) [39]. Two thousand highly variable genes were selected for PCA, batch effects were corrected with Harmony [40], and the top 20 PCs were used for neighbor graph construction and UMAP embedding. Clusters were defined using the Leiden algorithm (resolution = 0.5). Marker genes for canonical cell-type annotation are listed in the Supplementary Information.

### Concordance analysis between bulk RNA-seq and ST

To assess concordance between bulk RNA-seq and spatial transcriptomics datasets, pseudo-bulk ST counts were generated and analyzed with DESeq2. Rank–Rank Hypergeometric Overlap (RRHO2) [41, 42] was then applied to evaluate gene regulation concordance between the two approaches.

### Nuclei isolation and sorting

Nuclei were isolated from the prefrontal cortex (PFC) of FAST and AL mice (n = 3 per group) using a previously published protocol [43] with minor modifications. The PFC was flash-frozen, and nuclei were separated on a Percoll gradient (27, 31, and 35% in buffer) (Cytiva, Uppsala, Sweden). Given the distinct methylation profiles of neuronal and non-neuronal populations [44, 45], nuclei were sorted by NeuN expression into NeuN⁺ (neuronal) and NeuN⁻ (non-neuronal) fractions using a BD FACSAria II (BD Biosciences, Franklin Lakes, NJ), yielding a total of 12 samples. DNA was subsequently extracted for methylation analysis. Full experimental details are provided in the Supplementary Information.

### DNA methylation analysis

Genome-wide DNA methylation was assessed using the Infinium Mouse Methylation BeadChip ( ~ 285,000 CpG sites; Illumina, San Diego, California). Raw IDAT files were processed with the minfi pipeline [46] and normalized using the noob method. After quality control, 212,072 probes were retained for downstream analysis. Differentially methylated positions (DMPs) were identified using the limma [35]. Principal component analysis (PCA) was performed on β values, and linear models were applied to assess group effects, with cell type included as a covariate when appropriate. Effect sizes were reported as partial eta squared (η²p). Over-representation analysis (ORA) was performed using the clusterProfiler, and pathway clusters were summarized with the aPEAR package [47]. Detailed probe filtering criteria, normalization steps, and statistical parameters are provided in the Supplementary Information.

### Statistical analysis

For non-bioinformatic analyses, statistical tests were performed in GraphPad Prism version 10.6.0 (GraphPad Software, San Diego, CA, USA). Data are presented as mean ± SEM. Comparisons between two groups were assessed using two-tailed Student’s *t*-tests; when variances were unequal, Welch’s *t*-test was applied. Outcomes involving two or more factors were evaluated using two-way or three-way ANOVA to assess main effects and Sex × Treatment interactions, followed by the Holm-Sidak multiple comparisons test. Statistical significance was set at *p* < 0.05.

## Results

### Establishment of the maternal fasting model and maternal/offspring metabolic outcomes

**Fig. 1:**
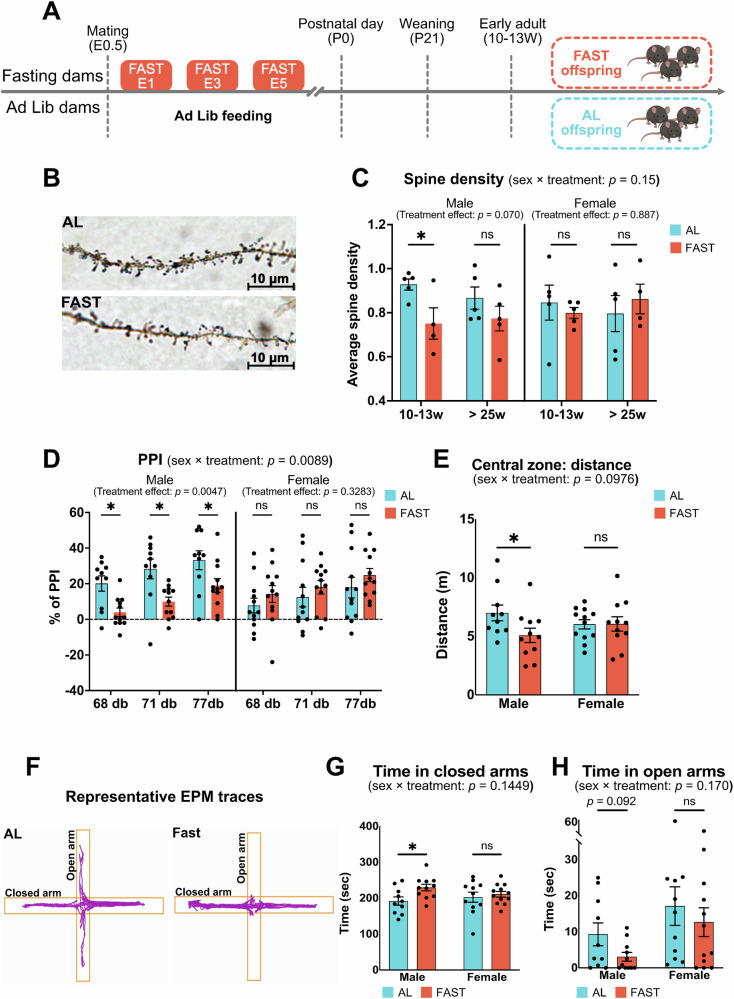
Maternal fasting paradigm and schizophrenia-like phenotypes in offspring. (**A**) Experimental design showing maternal fasting during early gestation. Created with BioRender.com. (**B**) Representative Golgi-stained dendrites of FAST male offspring (early adult). (**C**) Quantification of spine density (n = 4-5 mice per group; two-way ANOVA, main effect of treatment: *p* = 0.070; Holm-Sidak post hoc test, *p < 0.05). The Sex × Treatment interaction was evaluated using three-way ANOVA. (**D**) Prepulse inhibition (PPI) performance (n = 10-12 mice per group; two-way ANOVA, main effect of treatment: *p* = 0.0047; Holm-Sidak post hoc test, *p < 0.05). The Sex × Treatment interaction was evaluated using three-way ANOVA. (**E**) Distance traveled within the center zone (n = 10-12 mice per group). Comparisons within each sex were assessed using unpaired two-tailed Student’s *t*-test (*p < 0.05). The Sex × Treatment interaction was evaluated using two-way ANOVA. (**F**) Representative elevated plus maze (EPM) trajectories. (**G, H**) Time spent in closed and open arms (n = 10-12 mice per group). Comparisons within each sex were assessed using Student’s *t*-test (*p < 0.05). The Sex × Treatment interaction was evaluated using two-way ANOVA.

To model maternal undernutrition during early gestation, pregnant dams were subjected to 16-h fasting on embryonic days (E)1, E3, and E5, while being fed *ad libitum* (AL) on all other days (Fig. 1A). Pregnant dams subjected to the fasting paradigm, as well as their offspring, are hereafter collectively referred to as the maternal fasting group (FAST). Total maternal food intake across pregnancy did not differ between FAST and AL groups (Suppl. Figure 1A–C). At E5.5, FAST dams exhibited significantly reduced blood glucose and insulin levels relative to AL controls, whereas these parameters were comparable between groups at later gestational stages (Suppl. Figure 2A, B). Other maternal metabolic measures, including total cholesterol, triglycerides, and cortisol (Suppl. Figure 2C–E), as well as offspring body weight, general appearance, locomotor activity, and metabolic parameters, remained unchanged in both males (Suppl. Figure 3A-E) and females (Suppl. Figure 4A-E).

These findings suggest that transient maternal fasting during early gestation primarily affected maternal glucose and insulin levels at E5.5 but did not lead to persistent metabolic syndrome–like phenotypes in offspring. Moreover, compensatory food intake during subsequent *ad libitum* recovery days allowed FAST dams to return to baseline levels of food consumption before the next fasting exposure, resulting in comparable cumulative food intake across pregnancy.

### Structural and behavioral phenotypes in offspring

To investigate potential structural remodeling, Golgi staining was performed on coronal sections from early adult (10-13 weeks) and aged adult ( > 25 weeks) offspring. We analyzed spine density on secondary apical dendrites of pyramidal neurons within the prelimbic (PL) and infralimbic (IL) subregions of the mPFC (Suppl. Figure 5). For each animal, spine density was quantified across multiple dendritic segments, and the mean spine density per animal was used for all statistical analyses. Although the main effect of treatment was marginal (*p* = 0.070), post hoc comparisons revealed a significantly reduced spine density in FAST males during early adulthood, but this effect was not observed at the later time point (Fig. 1B-C). Conversely, no significant alterations were observed in female offspring (Fig. 1C).

Behavioral testing further revealed abnormalities in FAST males. In the prepulse inhibition (PPI) test, FAST males exhibited impaired sensorimotor gating, with significantly reduced PPI ratios at 68 dB, 71 dB and 77 dB prepulse intensities (Fig. 1D). Notably, three-way ANOVA revealed a significant Sex × Treatment interaction (*p* = 0.0089), suggesting sex-specific deficits in sensorimotor gating.

In the open field test (OF), FAST males showed shorter distances and fewer entries in the central zone (Fig. 1E, Suppl. Figure 6A, B), whereas total distance traveled was unchanged (Suppl. Figure 6C). In the elevated plus maze (EPM), FAST males spent more time in the closed arms and showed a trend of reduced time in the open arms (Fig. 1F-H), consistent with increased anxiety-related behavior. Other behavioral assays are summarized in Suppl. Table 2. No significant behavioral differences were detected in females (Suppl. Table 3). The results of sex × treatment effects in behavioral tests are summarized in Suppl. Table 4.

Together, these findings indicate that male offspring are particularly vulnerable to early maternal fasting exposure, exhibiting both structural and behavioral abnormalities consistent with schizophrenia-related phenotypes.

### Bulk RNA-seq reveals gene- and pathway- level alterations

To examine transcriptional consequences of maternal fasting, we performed bulk RNA sequencing of the early-adult PFC for both male and female offspring (n = 6 per group), respectively, to align with the structural and behavioral phenotypic analyses.

**Fig. 2:**
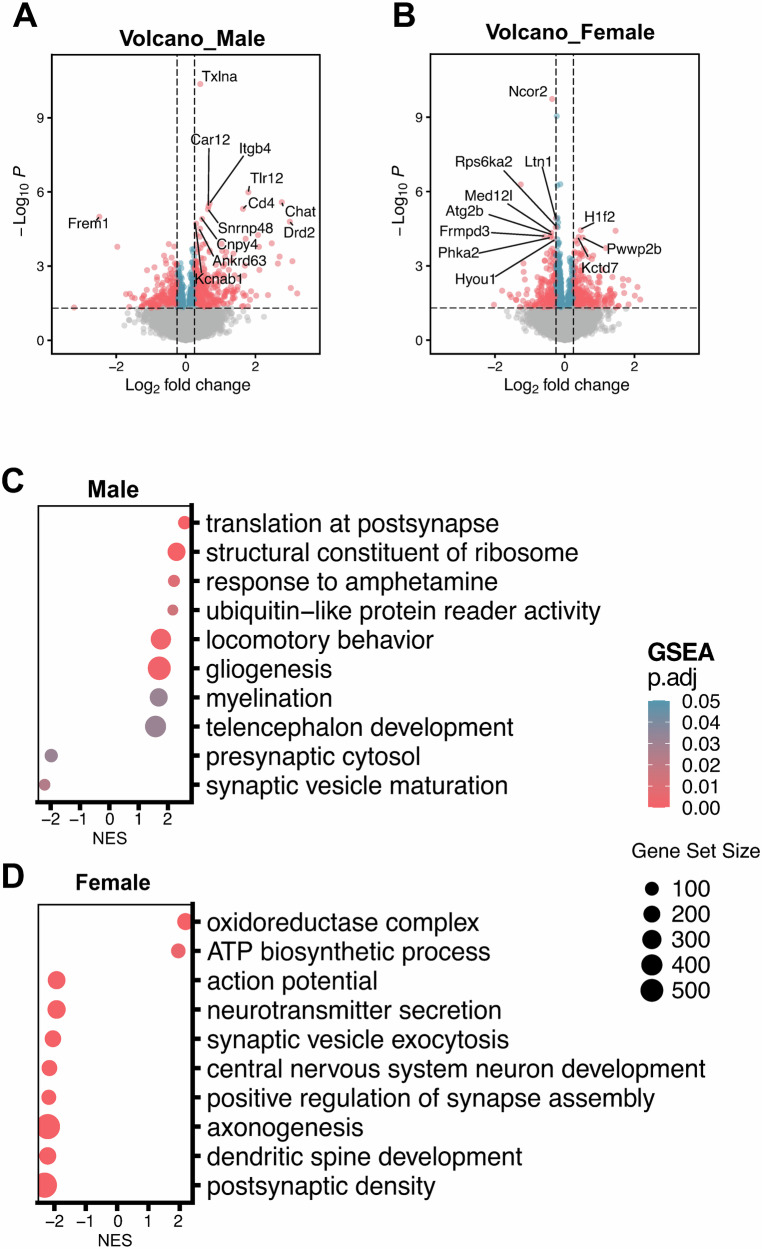
Bulk RNA-seq analysis. (**A**) Volcano plot showing differential gene expression in the mPFC of FAST male offspring, compared with AL male controls (n = 6 mice per group). Red dots indicate genes with nominal *p* < 0.05 and |log₂FC | > 0.25. Genes that remained significant after multiple testing correction (adjusted *p* < 0.05) are labeled. The y-axis represents -log_10_ (*p*-value). (**B**) Volcano plot showing differential gene expression in the mPFC of FAST female offspring, compared with AL female controls (n = 6 mice per group). Red dots indicate genes with nominal *p* < 0.05 and |log₂FC | > 0.25. Genes that remained significant after multiple testing correction (adjusted *p* < 0.05) are labeled. The y-axis represents -log_10_ (*p*-value). (**C**) Gene set enrichment analysis (GSEA) of male offspring showing the selected enriched pathways (FDR < 0.05). Dot size reflects gene set size, and dot color indicates adjusted *p*-value. NES, normalized enrichment score. (**D**) Gene set enrichment analysis (GSEA) of female offspring showing the selected enriched pathways (FDR < 0.05). Dot size reflects gene set size, and dot color indicates adjusted *p*-value. NES, normalized enrichment score.

For male offspring, differential expression analysis identified 12 differentially expressed genes (DEGs; BH-adjusted *p* < 0.05; |log₂FC | > 0.25) between FAST and AL groups, including *Txlna*, *Tlr12*, *Itgb4*, *Snrnp48*, *Chat*, *Car12*, *Cd4*, *Cnpy4*, *Frem1*, *Drd2*, *Kcnab1* and *Ankrd63* (Fig. 2A, Suppl. Table 5). For female offspring, 11 DEGs were identified (BH-adjusted *p* < 0.05; |log₂FC | > 0.25), including *Ncor2*, *Ltn1*, *Med12l*, *Frmpd3*, *Phka2*, *Hyou1*, *Rps6ka2*, *Atg2b*, *H1f2*, *Kctd7* and *Pwwp2b* (Fig. 2B, Suppl. Table 6).

Heatmaps of genes meeting nominal significance (*p* < 0.05) further illustrated group-wise shifts in expression profile in FAST males and females (Suppl. Fig. 7A, B).

Gene set enrichment analysis (GSEA) revealed significantly enriched pathways (FDR < 0.05) in both sexes.

For male offspring, positively enriched terms were related to synaptic processes, translation, and protein homeostasis, while negatively enriched pathways involved synaptic vesicle maturation and related presynaptic processes (Fig. 2C, Suppl. Table 7).

In females, pathways related to synaptic functions were negatively enriched, concurrent with positively enriched terms involving energy metabolism (Fig. 2D, Suppl. Table 8).

Together, these findings demonstrate that maternal fasting induced detectable transcriptional alterations in the PFC of offspring, which were associated with synaptic structural and functional processes highlighted by enrichment analyses.

### WGCNA identifies gene modules with sex-specific responses to maternal fasting

**Fig. 3:**
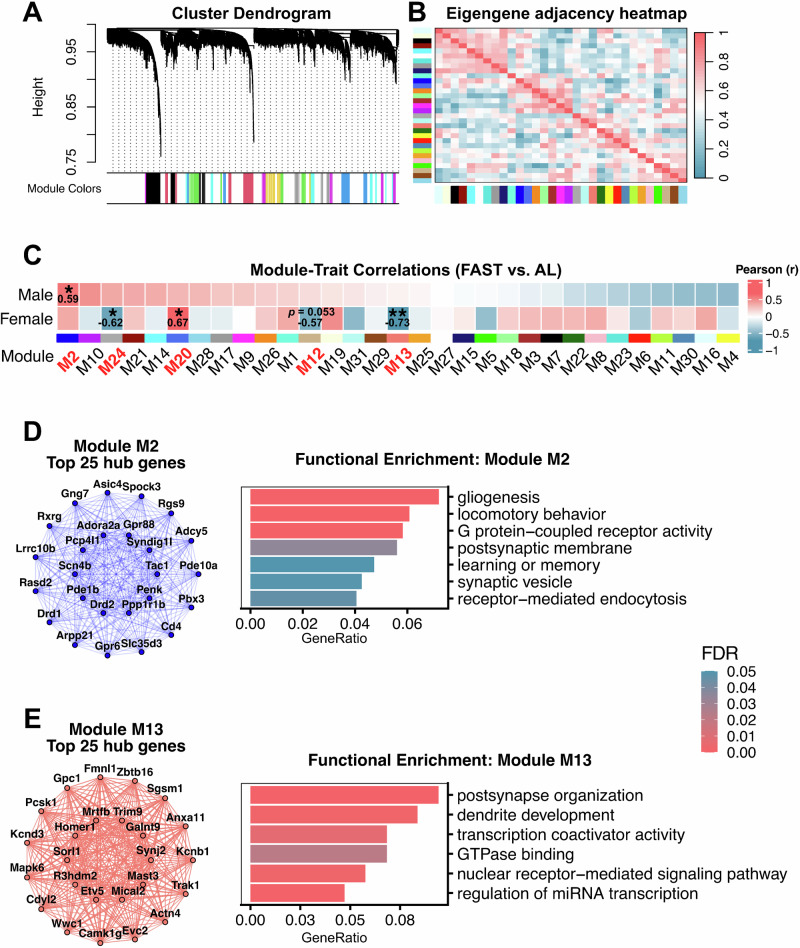
WGCNA identifies gene co-expression modules associated with maternal fasting exposure. (**A**) Dendrogram and module colors showing the co-expression modules identified by WGCNA. (**B**) Eigengene adjacency heatmap displaying the correlation matrix of module eigengenes. (**C**). Module-trait relationship heatmap showing the correlation between module eigengenes (MEs) and traits (FAST vs. AL) within each sex. The correlation coefficient and significance level (*p*-value) are labeled for selected modules (*p < 0.05, **p < 0.01). (**D**) Over-representation analysis (ORA) of module M2, showing selected significantly enriched pathways (FDR < 0.05) (right) and the network of the top 25 hub genes (left). Edges represent co-expression relationships between genes. (**E**) ORA of module M13, showing selected significantly enriched pathways (FDR < 0.05) (right) and the network of the top 25 hub genes (left). Edges represent co-expression relationships between genes.

To identify cross-sex gene modules responding to maternal fasting, we performed weighted gene co-expression network analysis (WGCNA) using sex-adjusted expression data from bulk RNA-seq of the PFC in both sexes. After net construction, a total of 31 co-expression modules were identified (Fig. 3A, B). Associations between eigengenes of each module and maternal fasting status (FAST–AL) were assessed using Pearson’s correlation coefficient (*r*), separately in male and female offspring (Fig. 3C, Suppl. Fig. 8A).

In male offspring, module M2 (blue) showed a strong positive correlation with maternal fasting exposure (*r* = 0.59, *p* = 0.046) (Fig. 3C). Over-representation analysis (ORA) indicated that M2 genes were enriched for pathways (FDR < 0.05) such as synaptic homeostasis and G protein-coupled receptor activity (Fig. 3D, Suppl. Table 9). Hub genes driving this module included *Drd2*, *Drd1*, *Ppp1r1b* and *Adora2a*.

For female offspring, module M13 (salmon) showed the strongest negative correlation with maternal fasting exposure (*r* = -0.73, *p* = 0.007) (Fig. 3C). This module, driven by hub genes such as *Homer1*, *Kcnb1*, and *Mapk6*, was enriched in synapse organization and transcription activity (Fig. 3E, Suppl. Table 9). Other significantly correlated modules (Suppl. Fig. 8B-D), including M24 (darkgrey; *r* = -0.62, *p* = 0.031) (Fig. 3C, Suppl. Fig. 8B) and M12 (tan; *r* = -0.57, *p* = 0.053) (Fig. 3C, Suppl. Fig. 8C), were enriched in functional terms related to synaptic membrane and developmental growth, respectively (Suppl. Fig. 8E-F, Suppl. Table 9). Moreover, module M20 (royalblue) represented the only positively correlated module in FAST females (*r* = 0.67, *p* = 0.018) (Fig. 3C, Suppl. Fig. 8D), it was driven by hub genes such as *Dusp5*, *Dusp6*, *Sik1*, and *Tet3*, whereas no significant pathway was revealed in the ORA results.

Collectively, we observed the upregulation of module M2 specifically in FAST male offspring, whereas females exhibited distinct module alterations. These findings highlight a sexually dimorphic transcriptional response to maternal fasting during early gestation.

### Regional transcriptomic alterations in the mPFC

**Fig. 4:**
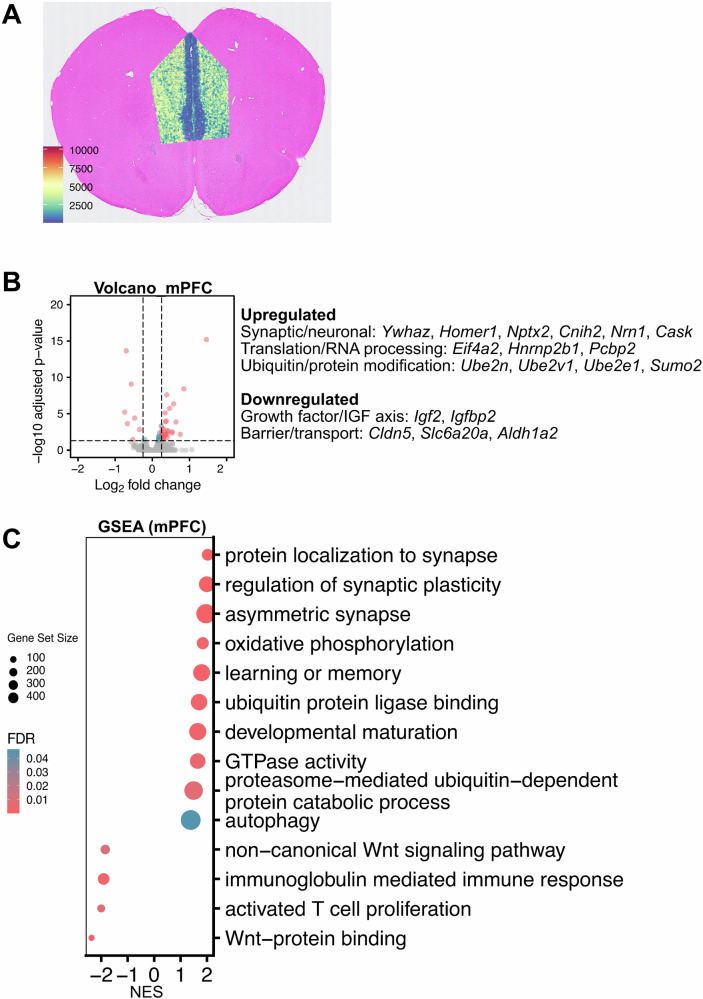
Regional alterations in the medial prefrontal cortex (mPFC) revealed by spatial transcriptomics. (**A**) Representative spatial transcriptomic section (sample AL1), with the mPFC region highlighted. Heatmap indicates total UMI counts per spot. (**B**) Volcano plot of differential expression analysis in the mPFC. Selected upregulated (e.g., *Ywhaz*, *Homer1*, *Nptx2*) and downregulated (e.g., *Igf2*, *Igfbp2*, *Cldn5*) genes are labeled. (**C**) Gene set enrichment analysis (GSEA) of the mPFC transcriptomic profile, showing selected significantly enriched pathways (FDR < 0.05). NES, normalized enrichment score.

To address the spatial constraints of bulk RNA-seq, we performed Visium HD spatial transcriptomics (ST) on two coronal PFC sections from each group of male offspring (Fig. 4A, Suppl. Fig. 9A). This allowed us to examine regional transcriptional alterations within the mPFC using pseudo-count matrices. A total of 83 DEGs were identified (70 upregulated, 13 downregulated) (Suppl. Table 10). Upregulated genes included those related to synaptic structure/plasticity (*Ywhaz*, *Homer1*, *Nptx2*, *Cnih2*, *Nrn1*, and *Cask*), protein translation/mRNA processing (*Eif4a2*, *Hnrnpa2b1*, and *Pcbp2*), and ubiquitin-related processes (*Ube2n*, *Ube2v1*, *Ube2e1*, and *Sumo2*) (Fig. 4B). Gene set enrichment analysis (GSEA) identified 261 positively enriched pathways (FDR < 0.05) and 81 negatively enriched pathways (Suppl. Table 11). Positively enriched pathways were predominantly related to synapse- organization, spine morphogenesis, proteasomal degradation, autophagy, and oxidative phosphorylation, while negatively ones were mainly associated with immune system processes and Wnt signaling pathway (Fig. 4C). These results show that spatial mapping highlights the mPFC as a hotspot for transcriptional dysregulation, converging on synaptic and protein metabolic pathways.

### Cell type-specific transcriptomic alterations in the mPFC

**Fig. 5:**
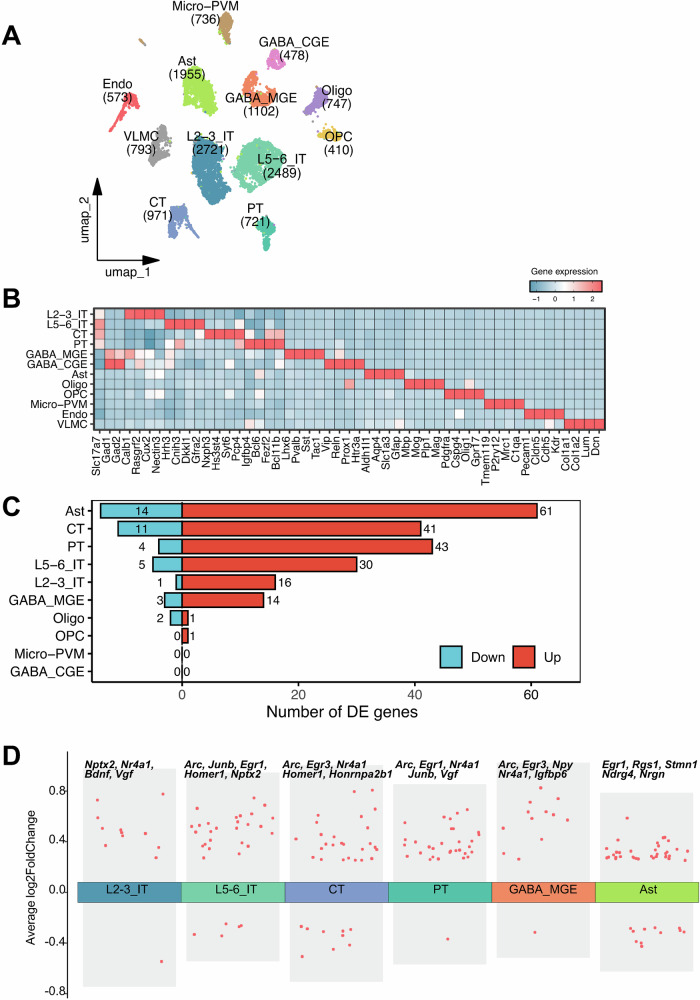
Cell type-specific spatial transcriptomic analysis. (**A**) UMAP projection of single-cell transcriptomic data from the mPFC, with major cell types annotated. Cell counts are indicated in parentheses. (**B**) Heatmap of representative marker genes used for cell-type identification across clusters. (**C**) Numbers of up- and down-regulated differentially expressed genes (DEGs) detected in each cell type (adjusted *p* < 0.05 and |log₂FC | > 0.25). (**D**) Cell type–specific DEGs in the mPFC. Representative genes (4-5 per cell type) are labeled for clarity; the full list of DEGs is provided in Suppl. Table 12. *Abbreviations*: IT, intratelencephalic; CT, corticothalamic; PT, pyramidal tract; GABA-MGE, medial ganglionic eminence–derived GABAergic neurons; GABA-CGE, caudal ganglionic eminence–derived GABAergic neurons; Ast, astrocytes; Oligo, oligodendrocytes; OPC, oligodendrocyte precursor cells; Micro-PVM, microglia/perivascular macrophages; VLMC, vascular and leptomeningeal cells.

To further resolve cell-type-level alterations, we generated single-cell transcriptome data from pooled male PFC tissue (n = 4 / group) using the 10x FLEX platform, yielding ~13,000 cells after quality control. Clustering analysis identified canonical neuronal and glial populations based on marker gene expression (Fig. 5A, B), which were then mapped to the ST data using RCTD for spot-level annotation (Suppl. Fig. 9B). Differential expression analysis of RCTD-deconvolved ST data revealed DEGs across multiple cell types, including astrocytes, corticothalamic (CT), pyramidal tract (PT), intratelencephalic neurons from deep layers (L5–6 IT) and superficial layers (L2/3 IT), and medial ganglionic eminence–derived GABAergic interneurons (GABA_MGE) (Suppl. Fig. 10A). Among these, astrocytes, CT, and PT neurons exhibited the largest numbers of DEGs. Most DEGs were upregulated in these cell types (Fig. 5C, Suppl. Table 12). Immediate-early genes such *Arc*, *Egr1*, *Egr3*, *Junb*, *Fos*, and *Nr4a1*, together with the ERK regulator *Dusp6*, were consistently elevated across neuronal subtypes (Fig. 5D). Over-representation analysis (ORA) of up-regulated DEGs highlighted pathways related to transcriptional regulation in neurons and synaptic function in astrocytes (Suppl. Fig. 10B-G, Suppl. Table 13). ORA of down-regulated DEGs did not yield robust enrichment; results are provided in Suppl. Table 14 for reference. Together, these findings pinpoint astrocytes and projection neurons as the principal cellular substrates of transcriptional changes induced by maternal fasting.

### Concordance between bulk RNA-seq and ST

To evaluate the concordance between the bulk RNA-seq and spatial transcriptomics data from male offspring, we generated pseudo-bulk ST counts and analyzed them with DESeq2 (Suppl. Table 15). To align with bulk RNA-seq, which included all PFC subregions, pseudo-bulk ST counts were generated across the entire PFC (mPFC, white matter, and surrounding cortex; Suppl. Table 15), whereas regional DEG lists restricted to the mPFC are provided in Suppl. Table 10. Rank–Rank Hypergeometric Overlap (RRHO2) analysis [41, 42] revealed strong concordance, with significant overlaps in both upregulated-upregulated and downregulated-downregulated quadrants (Suppl. Fig. 11A, B). Venn diagrams further depicted the overlap between genes classified as up- or down-regulated in both bulk RNA-seq and ST datasets. (Suppl. Fig. 11C-D). These results support that transcriptional alterations detected by bulk RNA-seq were robustly recapitulated in the ST data.

### Epigenetic remodeling in the PFC

To investigate how maternal fasting affected DNA methylation, we performed array-based profiling of NeuN⁺ (neuronal) and NeuN⁻ (non-neuronal) fractions isolated from the PFC of male offspring.

**Fig. 6:**
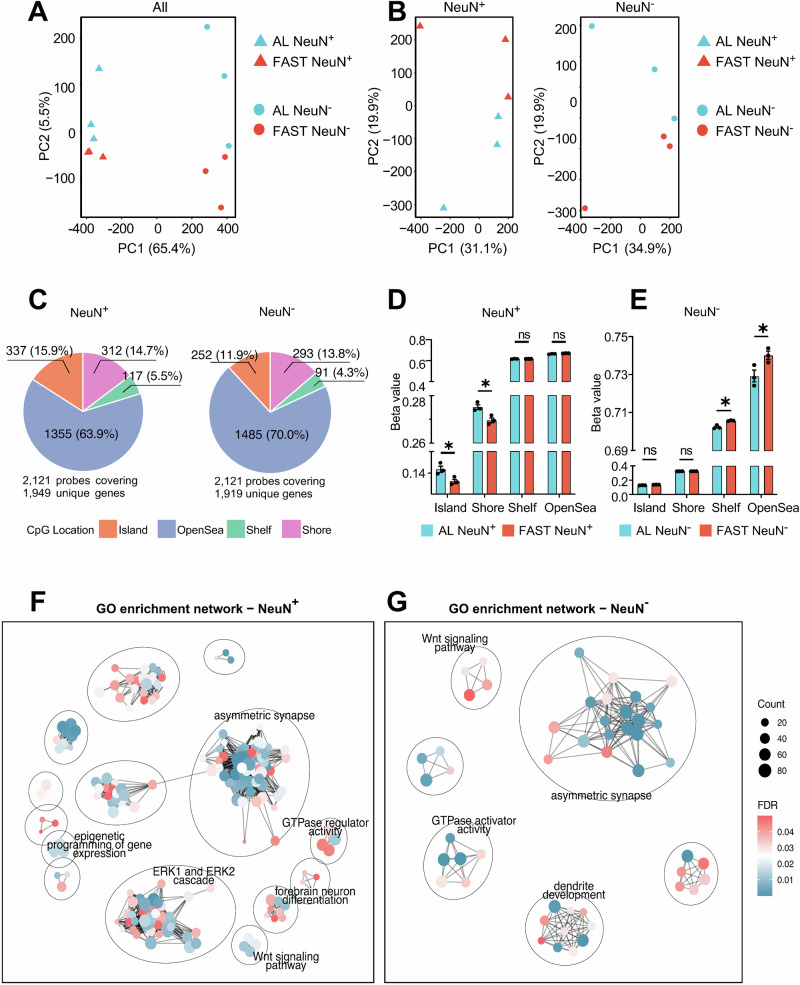
DNA methylation analysis. (**A**) Principal component analysis (PCA) of DNA methylation β values from across all NeuN⁺ and NeuN⁻ nuclei. (**B**) PCA of NeuN⁺ (left) and NeuN⁻ (right) nuclei analyzed separately. (**C**) Distribution of CpG locations among the top 1% of high-loading probes driving group separation in NeuN⁺ (left) and NeuN⁻ (right) dataset. (**D, E**) Average β values across CpG locations of the top 1% of high-loading probes in NeuN⁺ (D) and NeuN^-^ (E) datasets (*p < 0.05). (**F, G**) Over-representation analysis (ORA) of genes associated with high-loading probes in NeuN⁺ (**F**) and NeuN^-^ (**F**) dataset. Enriched pathways are grouped by functional similarity (Jaccard index and the Markov algorithm).

Principal component analysis (PCA) of all nuclei revealed a clear separation by cell type (PC1, *p* = 1.06e-9, η²*p* = 0.99) and a significant group effect (PC2, *p* = 0.01, η²*p* = 0.52) (Fig. 6A, Suppl. Table 16). When analyzed separately, PC2 significantly separated groups in NeuN⁺ nuclei (*p* = 0.042, η²*p* = 0.69) and showed a strong trend in NeuN⁻ nuclei (*p* = 0.057, η²*p* = 0.64) (Fig. 6B, Suppl. Table 16).

No individual CpG reached significance after multiple testing correction (Suppl. Fig. 12A, B). Thus, we conducted an exploratory PCA-loading-based analysis, in which the top 1% of probes contributing most strongly to group separation (PC2) were selected for downstream analyses (Fig. 6C, Suppl. Table 17).

In NeuN⁺ nuclei, these high-loading probes displayed hypomethylation at CpG islands and shores in FAST males, whereas in NeuN⁻ nuclei, high-loading probes exhibited hypermethylation at shelves and open sea regions (Fig. 6D-E). ORA revealed that high-loading probes in NeuN⁺ nuclei were enriched for pathways related to synapse organization, the ERK cascade, and Wnt signaling (Fig. 6F; Suppl. Table 18), whereas those in NeuN⁻ nuclei were enriched for pathways related to dendrite development and asymmetric synapse (Fig. 6G; Suppl. Table 18). These exploratory analyses suggest cell type–specific DNA methylation remodeling, linking neuronal hypomethylation and non-neuronal hypermethylation to synaptic and developmental pathways.

## Discussion

In this study, we established a novel schizophrenia model grounded in the DOHaD framework by mimicking acute famine exposure during early gestation. This developmental window coincides with global epigenomic reprogramming, a period of heightened vulnerability to environmental insults. By focusing on this critical stage, our study highlights how transient maternal malnutrition can leave enduring imprints on offspring brain development, providing a new perspective on the developmental origins of psychiatric disorders.

Alongside the schizophrenia-related phenotypic abnormalities observed in FAST male offspring, robust transcriptomic alterations were evident across multiple levels of analysis. Bulk RNA-seq revealed DEGs previously associated with schizophrenia (e.g., *Drd2* [48–50], *Cd4* [51–53], *Itgb4* [54], and *Kcnab1* [55]) and pathways related to synaptic function, translation, and proteostasis, whereas synaptic vesicle maturation was suppressed. Spatial transcriptomics of the mPFC further highlighted upregulation of synaptic plasticity genes (*Homer1* [56, 57], *Nptx2* [58, 59], and *Cask* [60, 61]) and enrichment in pathways related to protein homeostasis and autophagy. Conversely, downregulation of *Igf2* and *Igfbp2* suggested disrupted neurodevelopment [62]. Collectively, these results indicate that maternal fasting perturbs both synaptic organization and protein homeostasis in the PFC of FAST male offspring.

Cell type-resolved analyses provided additional mechanistic insights. Immediate-early genes (IEGs), including *Arc*, *Egr1*, *Egr3*, *Junb*, *Fos*, and *Nr4a1*, were consistently upregulated in excitatory neuronal subtypes, particularly in deep-layer neurons. These IEGs are regulated by multiple signaling pathways, including the MAPK/ERK cascade, which orchestrates activity-dependent transcription and synaptic remodeling [63–69]. Such maladaptive activation of IEG programs may contribute to the observed spine deficits and behavioral abnormalities, although further validation will be required.

Epigenetic remodeling provides a complementary layer of explanation that links transient maternal fasting to these long-term phenotypes. Exposure during early embryogenesis-a critical period of global DNA demethylation reprogramming-was associated with hypomethylation at CpG islands and shores in neurons, converging on synapse- and signal-related pathways. By contrast, non-neuronal cells exhibited hypermethylation in shelves and open sea regions, enriched for dendritic pathways. These cell type–specific methylation signatures parallel transcriptional alterations in bulk and spatial data, suggesting that nutritional stress reprograms the epigenome and thereby reinforces persistent transcriptional dysfunction.

From a pathophysiological perspective, a striking mismatch emerged: despite increased transcription and translation of synaptic proteins, spine density remained reduced in FAST male offspring. This suggests that excessively synthesized proteins were not incorporated into functional synapse but diverted to degradation pathways. The consistent upregulation of ubiquitin-related processes, together with enrichment of autophagy and oxidative phosphorylation, supports a scenario in which disrupted proteostasis and oxidative stress exacerbate vulnerability. Notably, dysregulation of pathways related to protein translation and degradation has also been reported in postmortem studies of schizophrenia [70–75], underscoring the translational relevance of our model.

While males were susceptible to early fasting exposure, female offspring did not exhibit overt phenotypic abnormalities. In WGCNA, Module M2, which is driven by critical schizophrenia-associated genes (e.g., *Drd2*, *Ppp1r1b* and *Adora2a*), was upregulated in FAST males, whereas it remained comparable between FAST and AL female offspring. Additionally, modules related to synaptic organization and transcriptional activities were downregulated in FAST females. These findings suggest a sex-specific transcriptional response to maternal fasting exposure and provide insight into the potential molecular mechanisms underlying the sex-dimorphic effects observed in our model.

In fact, sexually dimorphic outcomes have been reported in multiple models of prenatal environmental exposure, in which male offspring typically exhibit more severe phenotypic abnormalities [76–78]. Several hypotheses have been proposed, such as faster growth rates and greater requirements for nutritional support during early development, which render males more susceptible to nutritional challenges than females [79]. Moreover, sexually dimorphic DNA methylation patterns and placental function have been implicated in sex-specific vulnerability [80–82]. However, further studies will be required to elucidate the precise contribution of these mechanisms to the sex-based effects of prenatal exposure.

Beyond our prior lipid-restriction model [27], other maternal malnutrition models-including protein restriction and micronutrient deficiency-have also been shown to induce schizophrenia-related outcomes, such as impaired PPI, anxiety-like behavior, reduced spine density, and altered DNA methylation [83–93]. These models often differ in the timing of prenatal exposure (e.g., entire gestation in protein restriction models) and/or in the specific nutrients targeted (e.g., folate or vitamin B12 involved in one-carbon metabolism).

Importantly, unlike many DOHaD-based models that report long-term metabolic consequences resembling metabolic syndrome [22–26], our paradigm did not induce persistent metabolic abnormalities in the offspring. One possible explanation is the restricted timing of fasting (E1, E3, and E5), which corresponds to the peri-implantation period. At this very early stage, nutritional stress may preferentially affect epigenomic reprogramming and neural development rather than metabolic programming [94, 95]. This divergence further emphasizes the unique value of our model for studying psychiatric outcomes within the DOHaD framework.

Finally, in light of the DOHaD theory, the developmental plasticity induced by maternal fasting could represent a adaptive developmental strategy to prepare offspring for predicted postnatal malnutrition [96, 97]. Therefore, future studies are warranted to test whether postnatal food restriction, such as intermittent fasting, may ameliorate the defective phenotypes observed in FAST male offspring.

Several limitations should be acknowledged. First, our fasting paradigm was confined to the early embryonic period; its precise correspondence to stages of human pregnancy remains uncertain.

Second, because spine density analysis was conducted at the level of individual dendrites, the overall proportion of affected dendrites across entire brain sections could not be quantitatively determined.

Third, given the extensive remodeling of the central nervous system during development, whether the transcriptional alterations observed in FAST adulthood represent a direct continuation of embryonic changes remains to be elucidated.

Fourth, as phenotypic, transcriptomic, and epigenetic analyses were performed in separate cohorts of mice, direct correlation analyses across these assays were not feasible.

Fifth, this study focused exclusively on the mPFC; however, potential alterations in other brain regions warrant further investigation.

Sixth, while transcriptomic and epigenomic findings were strongly correlated, causal relationships between specific methylation changes and altered gene expression remain to be established.

Seventh, fasting represents a global reduction in all nutrients, leaving unresolved which specific factors are most critical. Finally, although we applied bulk, spatial, and single-cell analyses to characterize the PFC, cell–cell interactions within this region remain incompletely understood and warrant future investigation.

In summary, we present a novel DOHaD-based schizophrenia model demonstrating that transient maternal fasting during early embryogenesis reshapes the epigenome, induces broad transcriptional alterations, and disrupts synaptic organization and proteostasis. These findings highlight how acute nutritional stress during this critical window leaves lasting molecular and structural imprints on the brain, providing new insights into the developmental origins of schizophrenia.

## Supplementary information

Supplementary Information

Supplementary Table 1–11

Supplementary Table 12

Supplementary Table 13

Supplementary Table 14

Supplementary Table 15–18

## Data Availability

The raw data obtained in this study are available on DDBJ database (http://www.ddbj.nig.ac.jp/index-e.html) under the BioProject accession number PRJDB40357 (bulk RNA-seq, single-cell transcriptomes and DNA methylation) and PRJDB20747 (spatial transcriptomics). The corresponding BioSample metadata are available under accession numbers SAMD01818712-SAMD01818723 (bulk RNA-seq, male offspring), SAMD01818700-SAMD01818711 (bulk RNA-seq, female offspring), SAMD01695265 (single-cell transcriptomes), SAMD00912252–SAMD00912255 (spatial transcriptomics) and SAMD01688625–SAMD01688636 (DNA methylation). All scripts used for data processing and analysis are available on GitHub: https://github.com/HonboW77/Maternal-fasting.
