# Supplementary material for: Maternal fasting during early gestation induces epigenetic alterations and schizophrenia-related phenotypes
Source: Mol Psychiatry. 2026 May 13;31(9):5431–44. doi: 10.1038/s41380-026-03629-w (PMC13441970; doi:10.1038/s41380-026-03629-w)
Supplement: Supplementary file 1 [file 41380_2026_3629_MOESM1_ESM.pdf]

Supplementary Information

Supplementary Materials

Metabolic analysis

Oral glucose tolerance test (OGTT)

OGTT was performed after overnight fasting. Blood glucose was measured from a tail incision using a glucometer (Accu-Chek Guide Me, Roche). After baseline measurement, mice were orally administered 20% sucrose (0.05 mL/10 g body weight), and glucose levels were recorded every 15 min for four time points.

Insulin tolerance test (ITT)

After at least one week of rest, the same mice were subjected to ITT. Following a 4–5 h fast, baseline glucose levels were measured from the tail incision. Mice were then injected intraperitoneally with insulin (0.1 U/mL, Wako, Japan) at 0.0075 U/10 g body weight, and glucose levels were recorded every 15 min for four time points.

Plasma collection and biochemical assays

For blood insulin, cortisol, and lipid profiling, mice were anesthetized with isoflurane, and blood was collected from the right atrium. Samples were centrifuged at 4 °C, 1800 rpm for 20 min, and plasma was separated and stored at −80 °C until analysis.

- Plasma insulin levels: LBIS Mouse Insulin ELISA Kit (FUJIFILM, Japan)
- Plasma cortisol: Cortisol ELISA Kit (ENZO Life Sciences)
- Plasma total cholesterol (TCHO) and triglycerides (TG): DRI-CHEM 7000 Veterinary Chemistry Analyzer (FUJIFILM, Japan) using v-TCHO-P and v-TG-P slides.

Golgi staining and dendritic spine analysis

Brains were dissected, post-fixed overnight at 4 °C in the same fixative then washed with PBS. Coronal sections (100 μm) were cut using a vibratome (VT1200, Leica, Wetzlar, Germany), Golgi staining was performed according to the manufacturer's instructions. Bright-field images were acquired with a BX61 microscope (Olympus, Tokyo, Japan), and dendritic length and spine counts were quantified using cellSens software (Olympus). To minimize variability near branch points, only dendrites longer than 70 μm were analyzed, and the proximal 50 μm of the primary apical trunk was excluded from spine counts [1, 2]. Spine density was calculated as the number of spines divided by the length of the analyzed dendritic segment. All analyses were performed by an observer blinded to group assignments.

Behavioral test battery

A behavioral test battery was conducted in the following order: open field test (OF), novel object recognition test (NORT), Y-maze, elevated plus maze (EPM), marble burying test (MBT), tube test, forced swim test (FST), and prepulse inhibition (PPI). The OF, NORT, EPM, and FST sessions were video recorded. Animal behaviors were tracked and analyzed with ANY-maze software (v7.34, Stoelting Co., Wood Dale, IL, USA).

Prepulse inhibition test

The prepulse inhibition (PPI) test was conducted using SR-LAB startle systems (San Diego Instruments, San Diego, CA, USA) placed in a sound-attenuating chamber. Mice were acclimated for 5 min with 65 dB broadband background noise, followed by pseudo-randomized trials consisting of no-stimulus trials, pulse-alone trials (120 dB,

40 ms), and prepulse–pulse trials (20 ms prepulse at 68, 71, or 77 dB, presented 100 ms before the pulse). Percent PPI was calculated as 100 × (S – PP_i) / S, where S is the mean startle amplitude in pulse-alone trials and PP_i is the mean amplitude in prepulse–pulse trials at each prepulse intensity.

Open field test

Mice were placed in the center of an open-field arena (50 × 50 × 30 cm, L × W × H; grey acrylic walls) and allowed to explore freely for 10 min. Total distance traveled, time and distance spent in the central zone (25 × 25 cm, L × W), and the number of entries into the central zone were recorded and analyzed using ANY-maze software (v7.34, Stoelting Co., Wood Dale, IL, USA).

Elevated plus maze test

The elevated plus maze (EPM) consisted of two opposing open arms (25 × 5 cm, L × W) and two opposing closed arms (25 × 5 × 16 cm, L × W × H) connected by a central platform (5 × 5 cm, L × W). The apparatus was elevated 50 cm above the floor and constructed from grey acrylic, with opaque walls for the closed arms. Each mouse was placed on the central platform facing an open arm and allowed to explore freely for 5 min. The time spent in the open and closed arms and the number of entries into each arm were recorded and analyzed using ANY-maze software (v7.34, Stoelting Co., Wood Dale, IL, USA).

Y maze test

The Y-maze consisted of an equilateral triangular central zone connected to three identical arms (30 × 10 × 20 cm, L × W × H) positioned at 120° apart. Mice were placed

in the central zone and allowed to explore freely for 7 min. Correct alternations and total arm entries were automatically counted using ANY-maze software. The alternation rate was calculated as: Alternation % = (Number of correct alternations) / (Total entries – 2) × 100%. Total distance traveled was also analyzed.

Novel object recognition test (NORT)

NORT was performed over 5 consecutive days.

- Days 1–3 (habituation): mice free explored the empty open-field apparatus.
- Day 4 (NORT1): two identical objects were placed in adjacent corners of the arena; mice explored for 10 min.
- Day 5 (NORT2): one familiar object was replaced with a novel object; exploration lasted 10 min.

Exploration was defined as the mouse's nose being within 25 mm of the object. Time spent exploring familiar and novel objects was quantified with ANY-maze software. The discrimination index was calculated as: Discrimination % = [(Time_novel – Time_familiar) / (Time_novel + Time_familiar)] × 100%.

Marble burying test (MBT)

Mice were placed in transparent polypropylene cages (30 × 18 × 12 cm, L × W × H) filled with 5 cm of white paper bedding (SLC Inc., Japan). After a 30-min acclimation period, 15 glass marbles were arranged in a 3 × 5 grid on freshly renewed bedding. Mice were allowed to interact with the marbles for 30 min. Marbles covered by at least two-thirds were counted as buried.

Tube test

The tube test was performed only in male offspring using a transparent Plexiglas tube (33 mm internal diameter, 35 cm length). A clear divider separated the two mice initially. One *ad libitum* (AL) male and one FAST male offspring were simultaneously introduced from opposite ends; after removing the divider, the trial began.

- The mouse that retreated first (≥ 3 limbs outside the tube) was designated the loser.
- If neither withdrew, the trial was repeated.
- If three consecutive ties occurred, no winner was assigned.

Each mouse performed 10–12 trials. Winning rate was calculated as: Wins % = (Total wins) / (Total trials) × 100%.

Training trials were conducted a few days before the formal test.

Forced swim test (FST)

The FST was performed in a transparent acrylic cylinder (10 cm internal diameter, 24 cm height) filled with water (24 ± 1 °C, depth 20 cm), preventing mice from touching the bottom with tails or hind limbs. Each mouse was tested individually for 6 min. The first 2 min were considered acclimation and excluded. Immobility time during the last 4 min was recorded and analyzed with ANY-maze software.

Bulk RNA-seq analysis

Mice were anesthetized by inhalation of isoflurane (FUJIFILM Wako Pure Chemical Corporation, Osaka, Japan). Briefly, mice were placed in an anesthesia chamber containing a paper pad soaked with isoflurane until deep anesthesia was achieved. During the procedure, anesthesia was maintained using a nose cone. Mice were then decapitated, and brains were rapidly dissected on ice. The PFC was flash-frozen in liquid nitrogen and stored at −80 °C until use. Total RNA was extracted using the

RNeasy Plus Mini Kit (QIAGEN, Hilden, Germany) according to the manufacturer's protocol. Libraries were prepared using the SMART (Switching Mechanism at the 5' End of RNA Template) technology. Adapter sequences were appended to both ends of the first-strand cDNA, followed by PCR amplification. PCR products were purified with AMPure XP beads (Beckman Coulter), yielding double-stranded cDNA. Indexed sequencing libraries were then generated using adapter-recognizing primers with unique barcodes. The libraries were sequenced on the Illumina NovaSeq X Plus platform (San Diego, California).

Visium HD ST data analysis

Mice were anesthetized by inhalation of isoflurane (FUJIFILM Wako Pure Chemical Corporation, Osaka, Japan). Briefly, mice were placed in an anesthesia chamber containing a paper pad soaked with isoflurane until deep anesthesia was achieved. During the procedure, anesthesia was maintained using a nose cone, and mice were transcardially perfused with 4% paraformaldehyde (PFA) in Milli-Q water, followed by overnight post-fixation at 4 °C. Brains were processed into FFPE blocks by dehydration, xylene clearing, and paraffin embedding. Coronal sections (5 μm) of the prefrontal cortex (Bregma +1.98 to +1.94 mm) were cut and mounted on glass slides. The following section process and HE staining as well as the library construction were performed according to the manufacturer's instructions Visium HD FFPE Tissue Preparation Handbook (CG000684) and Visium HD Spatial Gene Expression Reagent Kits User Guide (CG000685).

16-micrometer binned outputs generated by Space Ranger version 3.1.2 pipeline were used for analysis. The medial prefrontal cortex (mPFC) region was manually delineated on ST images of each sample using the napari_spatialdata package

[3] in Python 3.12. Data were then converted into Seurat objects for downstream analysis and visualization.

For quality control, spots with total UMI counts or numbers of detected genes below the 1st percentile of the data, or with mitochondrial read fractions > 10%, were excluded. Genes detected in fewer than 50 spots were also removed. Data were log1p-normalized and scaled to 10,000 per spot using Seurat's NormalizeData function. Single-cell based spatial deconvolution was performed using the RCTD package [4] with default parameters, leveraging annotated single-cell reference profiles for spot-level cell type assignment.

Differential expression analysis was performed either by generating pseudo-bulk count matrices for targeted regions and analyzed with DESeq2, or by applying the FindMarkers function in Seurat with the MAST method, including nCount_Spatial as a latent variable. Gene set enrichment analysis (GSEA) [5] and over-representation analysis (ORA) were conducted with the clusterProfiler package [6], with genes included in differential analysis as the universe.

Library construction and quality control for 10X FLEX single-cell data

For each group, pooled samples were fixed with formaldehyde and dissociated. Target probes from the Chromium Fixed RNA Profiling kit (10x Genomics) were hybridized to intracellular RNA and ligated to join adjacent probe pairs. To equalize input cell numbers, specimens were mixed accordingly before encapsulation. Cells and barcoded primer beads were co-encapsulated on the Chromium X instrument to generate Gel Beads-in-Emulsion (GEMs). Within droplets, probe–primer annealing and extension appended cell-identifying adapter sequences to the ligated probes. Indexed PCR was then performed to amplify libraries, which were purified and prepared for sequencing.

FASTQ files were processed with Cell Ranger v9.0.1 using default settings for Next GEM Flex Gene Expression Multiplex, generating raw and filtered feature–barcode matrices. Cell calling was performed on raw count matrices from the Cell Ranger pipeline using EmptyDrops [7]; droplets with < 15 UMIs were considered empty, and barcodes with FDR < 0.001 were retained. Probes with predicted off-target activity were excluded. Ambient RNA contamination was estimated and corrected with SoupX [8]. The soup profile was calculated from raw matrices with default parameters, and the contamination fraction was manually set to 0.2 before adjustment.

For quality control (QC), genes detected in fewer than 20 cells were discarded. Multiplets were identified with scDblFinder [9]. Cells were retained if nCount > 500, nFeature > 400, and mitochondrial reads < 5%. Characterized by high mitochondrial signatures, as well as residual doublet clusters, were excluded from downstream analysis. After QC, 13,696 cells (7,812 ad libitum; 5,884 FAST) were retained.

Marker genes used for canonical cell types of annotation

Differential expression for each cell type was performed using the FindAllMarkers function in Seurat, with marker genes referenced from the Allen Brain Map (Mouse - Whole cortex & hippocampus 10x; https://portal.brain-map.org/) [10]. Neurons were first classified into excitatory (*Slc17a7*) and inhibitory (*Gad1*, *Gad2*) populations. Excitatory neurons were further subdivided into L2/3 intratelencephalic (IT; *Calb1*, *Rasgrf2*, *Cux2*, *Nectin3*), L5 IT (*Hrh3*, *Cnih3*, *Dkkl1*, *Gfra2*), corticothalamic (CT; *Nxph3*, *Hs3st4*, *Syt6*, *Pcp4*), pyramidal tract (PT; *Igfbp4*, *Bcl6*, *Fezf2*, *Bcl11b*) subtypes. Inhibitory neurons were categorized by developmental origin, with medial ganglionic eminence (MGE) markers including *Sst*, *Pvalb*, *Lhx6*, *Tac1*, and caudal ganglionic eminence (CGE) markers including *Vip*, *Reln*, *Prox1*, *Htr3a*. Astrocytes Glial

populations were identified by canonical markers as follows: astrocytes (Ast; *Aldh1l1*, *Aqp4*, *Slc1a3*, *Gfap*), oligodendrocytes (Oligo; *Mbp*, *Mag*, *Mog*, *Plp1*), oligodendrocyte precursor cells (OPCs; *Pdgfra*, *Cspg4*, *Olig1*, *Gpr17*), and microglia/perivascular macrophages (Micro-PVM; *Tmem119*, *P2ry12*, *Mrc1*, *C1qa*). Endothelial cells were characterized by expression of *Pecam1*, *Cldn5*, *Cdh5*, *Kdr*, while vascular and leptomeningeal cells (VLMCs) were identified with *Col1a1*, *Col1a2*, Lum, *Dcn*.

Concordance analysis between bulk RNA-seq and ST dataset

Differential expression analysis of ST data was performed on pseudo-bulk count matrices generated from each sample using DESeq2, and then compared with the bulk RNA-seq results using the R package RRHO2 [11, 12], with the multiple testing parameter set to BH correction. Heatmaps were generated with RRHO2_heatmap function, and quadrant hotspots were interpreted as follows: upper left, genes up-regulated in ST and down-regulated in bulk RNA-seq; upper right, genes down-regulated in both datasets; lower left, genes up-regulated in both datasets; lower right, genes down-regulated in ST and up-regulated in bulk RNA-seq (Suppl. Fig. 11A). Venn diagrams showing overlapping genes were generated with RRHO2_vennDiagram function.

Nuclei isolation and sorting

Nuclei were isolated from the prefrontal cortex (PFC) based on a previously published protocol [13] with minor modifications. The PFC was flash-frozen in liquid nitrogen and stored at −80 °C until use. Tissue homogenate was loaded onto a discontinuous Percoll gradient (27%, 31%, and 35%) and centrifuged at 40,200 × g for 10 min at 4 °C in an Himac CP80NX ultracentrifuge (rotor: P50A3, Hitachi, Ibaraki, Japan). Nuclei

collected from the 31%/35% interface were blocked with 0.5% BSA for 30 min at 4 °C and incubated with Alexa Fluor 488–conjugated NeuN antibody (1:1000, Millipore, cat. MAB377X) for 2 hours at 4 °C. Sorting was performed on a BD FACSAria II (BD Biosciences, Franklin Lakes, NJ), and $NeuN^+$ and $NeuN^-$ fractions were collected separately, yielding a total of 12 samples. DNA was extracted using the QIAamp UCP DNA Micro Kit (QIAGEN, Hilden, Germany) following the manufacturer's instructions.

DNA methylation analysis

Raw IDAT files were processed with the minfi pipeline [14] and normalized using the noob method. Probe annotation (gene names, CpG locations) was mapped to a customized Illumina Mouse Methylation manifest (mouse mm10 genome), yielding β values, detection *p*-values, and methy/unmethylated intensities. CpG locations were consolidated such that N_Shore and S_Shore were grouped as "Shore," and N_Shelf and S_Shelf as "Shelf."

M-values were calculated as $\log_2$[(methylated intensity + 100) / (unmethylated intensity + 100)] [15]. Probes were excluded if they lacked gene annotation, had a detection $p > 0.01$ in any sample, or mapped to multiple CpG locations, leaving 212,072 probes for analysis.

Differentially methylated positions (DMPs) were identified from M-values using the limma with empirical Bayes–moderated t-tests [16]. Principal component analysis (PCA) was performed on β values (prcomp in R), and associations between PCs and experimental factors were tested using linear models. In the combined dataset ($NeuN^+$ and $NeuN^-$), group was modeled with cell type as a covariate; subset analyses included group only. Factor effects were tested with drop-one F tests, and partial eta

squared (η^2p) were reported as effect sizes. ORA was performed with clusterProfiler, using all genes in the analyzed dataset as the universe. Pairwise similarity of enriched pathways was calculated using the Jaccard index, and pathway clusters were identified with the Markov algorithm implemented in the aPEAR [17].

References

1. Maekawa M, Ohnishi T, Toyoshima M, Shimamoto-Mitsuyama C, Hamazaki K, Balan S, et al. A potential role of fatty acid binding protein 4 in the pathophysiology of autism spectrum disorder. Brain Commun. 2020;2:fcaa145.
2. Wada Y, Maekawa M, Ohnishi T, Balan S, Matsuoka S, Iwamoto K, et al. Peroxisome proliferator-activated receptor α as a novel therapeutic target for schizophrenia. eBioMedicine. 2020;62:103130.
3. Marconato L, Palla G, Yamauchi KA, Virshup I, Heidari E, Treis T, et al. SpatialData: an open and universal data framework for spatial omics. Nat Methods. 2025;22:58–62.
4. Cable DM, Murray E, Zou LS, Goeva A, Macosko EZ, Chen F, et al. Robust decomposition of cell type mixtures in spatial transcriptomics. Nat Biotechnol. 2022;40:517–526.
5. Subramanian A, Tamayo P, Mootha VK, Mukherjee S, Ebert BL, Gillette MA, et al. Gene set enrichment analysis: A knowledge-based approach for interpreting genome-wide expression profiles. Proc Natl Acad Sci. 2005;102:15545–15550.
6. Xu S, Hu E, Cai Y, Xie Z, Luo X, Zhan L, et al. Using clusterProfiler to characterize multiomics data. Nat Protoc. 2024;19:3292–3320.
7. Lun ATL, Riesenfeld S, Andrews T, Dao TP, Gomes T, Marioni JC, et al. EmptyDrops: distinguishing cells from empty droplets in droplet-based single-cell RNA sequencing data. Genome Biol. 2019;20:63.
8. Young MD, Behjati S. SoupX removes ambient RNA contamination from droplet-based single-cell RNA sequencing data. GigaScience. 2020;9:giaa151.
9. Germain P-L, Lun A, Garcia Meixide C, Macnair W, Robinson MD. Doublet identification in single-cell sequencing data using scDblFinder. F1000Research.

2022;10:979.

10. Yao Z, Liu H, Xie F, Fischer S, Adkins RS, Aldridge AI, et al. A transcriptomic and epigenomic cell atlas of the mouse primary motor cortex. Nature. 2021;598:103–110.
11. Plaisier SB, Taschereau R, Wong JA, Graeber TG. Rank–rank hypergeometric overlap: identification of statistically significant overlap between gene-expression signatures. Nucleic Acids Res. 2010;38:e169.
12. Cahill KM, Huo Z, Tseng GC, Logan RW, Seney ML. Improved identification of concordant and discordant gene expression signatures using an updated rank-rank hypergeometric overlap approach. Sci Rep. 2018;8:9588.
13. Bundo M, Kato T, Iwamoto K. Cell Type-Specific DNA Methylation Analysis in Neurons and Glia. In: Karpova N, editor. Epigenetic Methods Neurosci. Res., New York, NY: Springer; 2016. p. 115–123.
14. Aryee MJ, Jaffe AE, Corrada-Bravo H, Ladd-Acosta C, Feinberg AP, Hansen KD, et al. Minfi: a flexible and comprehensive Bioconductor package for the analysis of Infinium DNA methylation microarrays. Bioinformatics. 2014;30:1363–1369.
15. Du P, Zhang X, Huang C-C, Jafari N, Kibbe WA, Hou L, et al. Comparison of Beta-value and M-value methods for quantifying methylation levels by microarray analysis. BMC Bioinformatics. 2010;11:587.
16. Ritchie ME, Phipson B, Wu D, Hu Y, Law CW, Shi W, et al. limma powers differential expression analyses for RNA-sequencing and microarray studies. Nucleic Acids Res. 2015;43:e47–e47.
17. Kerseviciute I, Gordevicius J. *aPEAR* : an R package for autonomous visualization of pathway enrichment networks. Bioinformatics. 2023;39:btad672.

Supplementary Figures

Suppl. Fig. 1. Maternal food intake.

(A) Daily food intake of dams from embryonic days (E)1 to E8, and cumulative intake during E8–14 and E14–18.

(B) Cumulative food intake during early gestation (E1–8) in ad libitum (AL) and fasting (FAST) dams (n = 7 per group; ns, not significant).

(C) Total food intake across the entire gestational period (E1–18) (n = 7 per group; ns, not significant).

Suppl. Fig. 2. Maternal metabolism.

(A–D) Maternal blood glucose, plasma insulin, triglyceride (TG), and total cholesterol (TCHO) levels measured at E5.5 (end of fasting), E10.5, and E15.5 (n = 5 per group; *$p < 0.05$, **$p < 0.01$, ns, not significant).

(E) Maternal plasma cortisol levels measured at E5.5 (n = 5 per group; ns, not significant).

Suppl. Fig. 3. Offspring body weight and metabolism (male).

(A) Body weight trajectories of male offspring from 1 to 10 weeks of age (AL, n = 25–65; FAST, n = 17–33; two-way ANOVA, $p = 0.1076$ for group effect; Sidak post hoc test, ns = not significant).

(B, C) Plasma triglyceride (TG) and total cholesterol (TCHO) levels in male offspring (n = 10-12 per group; ns, not significant).

(D) Oral glucose tolerance test (OGTT) curves and corresponding area under the curve (AUC) in male offspring (n = 10–12 per group; ns, not significant).

(E) Insulin tolerance test (ITT) curves and corresponding AUC in male offspring (n =

10–12 per group; ns, not significant).

Suppl. Fig. 4. Offspring body weight and metabolism (female).

(A) Body weight trajectories of female offspring from 1 to 10 weeks of age (AL, n = 25–65; FAST, n = 17–33; two-way ANOVA, $p = 0.4660$ for group effect; Sidak post hoc test, ns = not significant).

(B, C) Plasma triglyceride (TG) and total cholesterol (TCHO) levels in female offspring (n = 10-12 per group; ns, not significant).

(D) OGTT curves and corresponding AUC in female offspring (n = 12 per group; ns, not significant).

(E) ITT curves and corresponding AUC in female offspring (n = 12 per group; ns, not significant).

Suppl. Fig. 5. Dendritic spine density analysis.

Brain regions selected for dendritic spine density analysis, focusing on the prelimbic (PL) and infralimbic (IL) areas of the medial prefrontal cortex. Analysis was performed on secondary apical dendrites of pyramidal neurons, excluding the proximal 50 µm segment.

Suppl. Fig. 6. Open-field test results in male offspring.

(A) Representative open-field (OF) trajectories of male offspring.

(B) Number of entries into the center zone in the OF test (n = 10-12 mice per group). Comparisons within each sex were assessed using Student's t-test ($^{**}p < 0.01$). The Sex × Treatment interaction was evaluated using two-way ANOVA.

(C) Total distance traveled in the OF test (n = 10-12 mice per group). Comparisons

within each sex were assessed using Student's t-test. The Sex × Treatment interaction was evaluated using two-way ANOVA.

Suppl. Fig. 7. Bulk RNA-seq analysis.

(A) Heatmap showing gene expression profiles of prefrontal cortex samples form ad libitum (AL) and fasting (FAST) male offspring. Genes with nominal $p < 0.05$ are displayed, and those with adjusted $p < 0.05$ are highlighted. Representative differentially expressed genes are indicated on the left.

(B) Heatmap showing gene expression profiles of prefrontal cortex samples from AL and FAST female offspring. Genes with nominal $p < 0.05$ are displayed, and those with adjusted $p < 0.05$ are highlighted. Representative differentially expressed genes are indicated on the left.

Suppl. Fig. 8. WGCNA module eigengenes (MEs) and co-expression modules.

(A). Full results of the correlation between module eigengenes (MEs) and traits (FAST vs. AL) within each sex. The correlation coefficient and significance level (p value) are labeled (*$p < 0.05$, **$p < 0.01$).

(B-D) Network visualization of the top 25 hub genes for modules M24, M12, and M20. Edges represent co-expression relationships between genes.

(E, F) Over-representation analysis (ORA) of modules M24 and M12, showing selected significantly enriched pathways (FDR < 0.05).

Suppl. Fig. 9. Spatial transcriptomics and cell-type deconvolution.

(A) Representative spatial transcriptomic sections from samples AL2, FAST1 and FAST2, with the medial prefrontal cortex (mPFC) highlighted. Color scale indicates

total UMI counts per spot.

(B) Cell type deconvolution results showing the spatial distribution of major cell types across each section.

Abbreviations: IT, intratelencephalic; CT, corticothalamic; PT, pyramidal tract; GABA-MGE, medial ganglionic eminence–derived GABAergic neurons; GABA-CGE, caudal ganglionic eminence–derived GABAergic neurons; Ast, astrocytes; Oligo, oligodendrocytes; OPC, oligodendrocyte precursor cells; Micro-PVM, microglia/perivascular macrophages; VLMC, vascular and leptomeningeal cells.

Suppl. Fig. 10. Cell type-specific DEGs in the mPFC and ORA of upregulated DEGs.

(A) Numbers of differentially expressed genes (DEGs) identified in each cell type of the mPFC (adj. $p < 0.05$ and $|\log_2 FC| > 0.25$).

(B–G) Over-representation analysis (ORA) of upregulated DEGs in L2-3 intratelencephalic (IT), L5-6 IT, corticothalamic (CT), pyramidal tract (PT), GABAergic neurons of medial ganglionic eminence origin (GABA-MGE), and astrocytes (Ast). The top enriched pathways for each cell type are shown.

Suppl. Fig. 11. Concordance of gene regulation between bulk RNA-seq and spatial transcriptomics (ST).

(A) Schematic illustration of the interpretation of four quadrants in the rank–rank hypergeometric overlap (RRHO) heatmap, indicating concordant (both upregulated or both downregulated) and discordant (opposite directions) gene regulation between ST and bulk RNA-seq.

(B) Rank–rank hypergeometric overlap (RRHO) stratified heatmap showing genome-

wide concordance of differential expression between bulk RNA-seq and ST datasets. Each pixel represents the -log10 (p-value) from a hypergeometric test, with quadrants indicating concordant or discordant overlaps.

(C, D) Venn diagrams illustrating the number of overlaps between up- or down-regulated genes in bulk RNA-seq and ST.

Suppl. Fig. 12. Differentially methylated position (DMP) analysis in $NeuN^+$ and $NeuN^-$ nuclei.

(A) Volcano plot of DMPs in $NeuN^+$ nuclei.

(B) Volcano plot of DMPs in $NeuN^−$ nuclei.

Supplementary Tables

Suppl. Table 1.

Number of litters used in major analyses.

Suppl. Table 2.

Summary of behavioral test results in male offspring exposed to maternal fasting.

Suppl. Table 3.

Summary of behavioral test results in female offspring exposed to maternal fasting.

Suppl. Table 4.

Summary of sex × treatment effects in behavioral tests.

Suppl. Table 5.

Differentially expressed genes (DEGs) identified by bulk RNA sequencing of the medial prefrontal cortex (mPFC) in male offspring exposed to maternal fasting.

Suppl. Table 6.

Differentially expressed genes (DEGs) identified by bulk RNA sequencing of the medial prefrontal cortex (mPFC) in female offspring exposed to maternal fasting.

Suppl. Table 7.

Gene set enrichment analysis (GSEA) of bulk RNA-seq data from the medial prefrontal cortex (mPFC) of male offspring exposed to maternal fasting.

Suppl. Table 8.

Gene set enrichment analysis (GSEA) of bulk RNA-seq data from the medial prefrontal cortex (mPFC) of female offspring exposed to maternal fasting.

Suppl. Table 9.

Over-representation analysis (ORA) of gene co-expression modules.

Suppl. Table 10.

Differentially expressed genes (DEGs) identified by spatial transcriptomics (ST) in the medial prefrontal cortex (mPFC) of male offspring exposed to maternal fasting.

Suppl. Table 11.

Gene set enrichment analysis (GSEA) of spatial transcriptomics (ST) data from the medial prefrontal cortex (mPFC) of male offspring exposed to maternal fasting.

Suppl. Table 12.

Differentially expressed genes (DEGs) identified by cell type–resolved analysis of RCTD-deconvolved spatial transcriptomics data.

Suppl. Table 13.

Over-representation analysis (ORA) of up-regulated DEGs across cell types in the PFC.

Suppl. Table 14.

Over-representation analysis (ORA) of down-regulated DEGs across cell types in the PFC.

Suppl. Table 15.

Pseudo-bulk ST differential expression results from the entire PFC (mPFC, white matter, and surrounding cortex) for concordance analysis with bulk RNA-seq.

Suppl. Table 16.

Principal component analysis (PCA) results of DNA methylation data from $NeuN^+$ (neuronal) and $NeuN^–$ (nonneuronal) nuclei.

Suppl. Table 17.

Top 1% CpG probes with the highest loading on PC2 identified by principal component analysis (PCA) of DNA methylation in $NeuN^+$ and $NeuN^-$ nuclei.

Suppl. Table 18.

Over-representation analysis (ORA) of high-loading CpG probes in $NeuN^+$ nuclei.

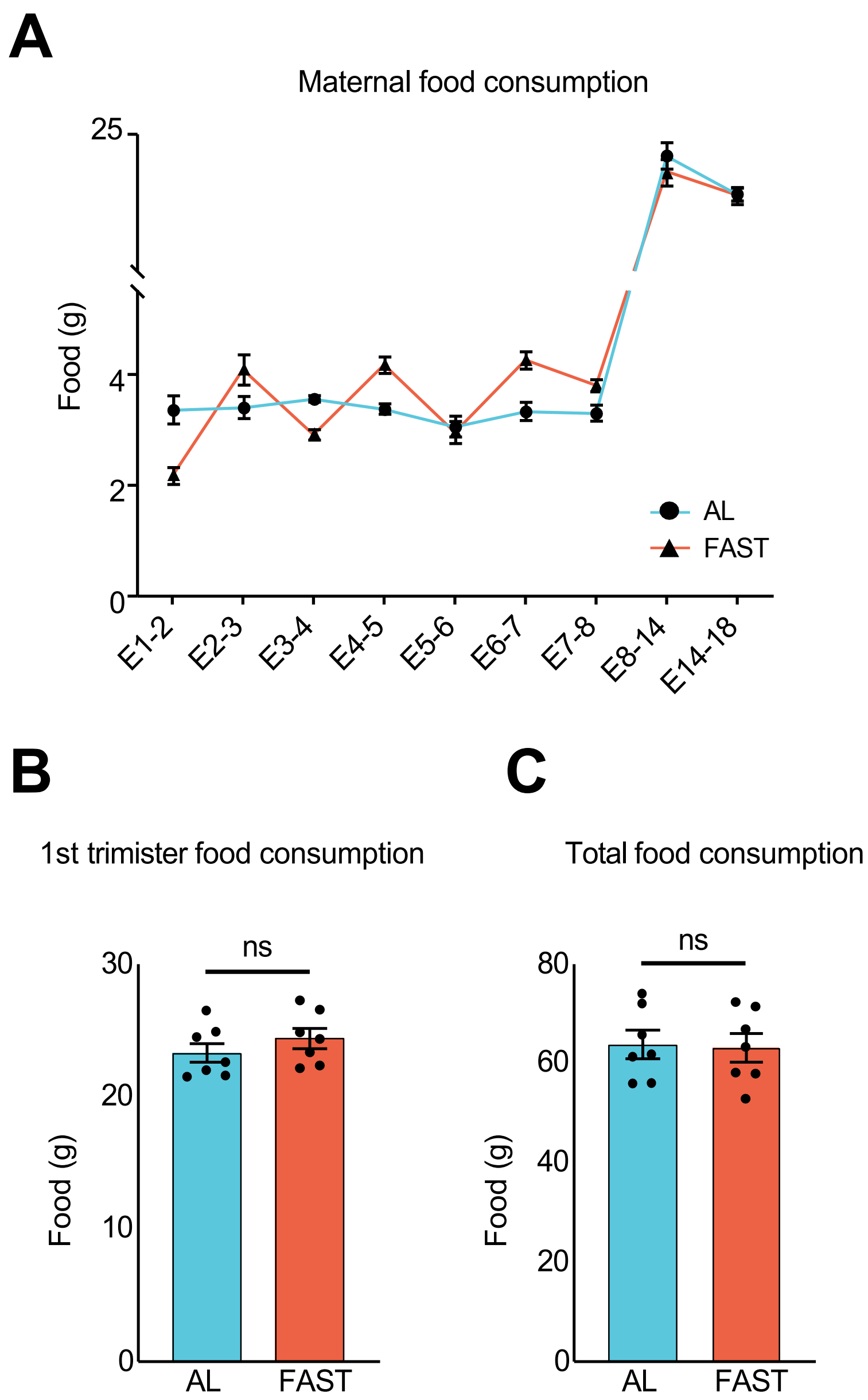

A
Maternal food consumption
25
4
2
0
Food (g)
AL
FAST
E1-2
E2-3
E3-4
E4-5
E5-6
E6-7
E7-8
E8-14
E14-18
B
1st trimister food consumption
ns
30
20
10
0
Food (g)
AL
FAST
C
Total food consumption
ns
80
60
40
20
0
Food (g)
AL
FAST

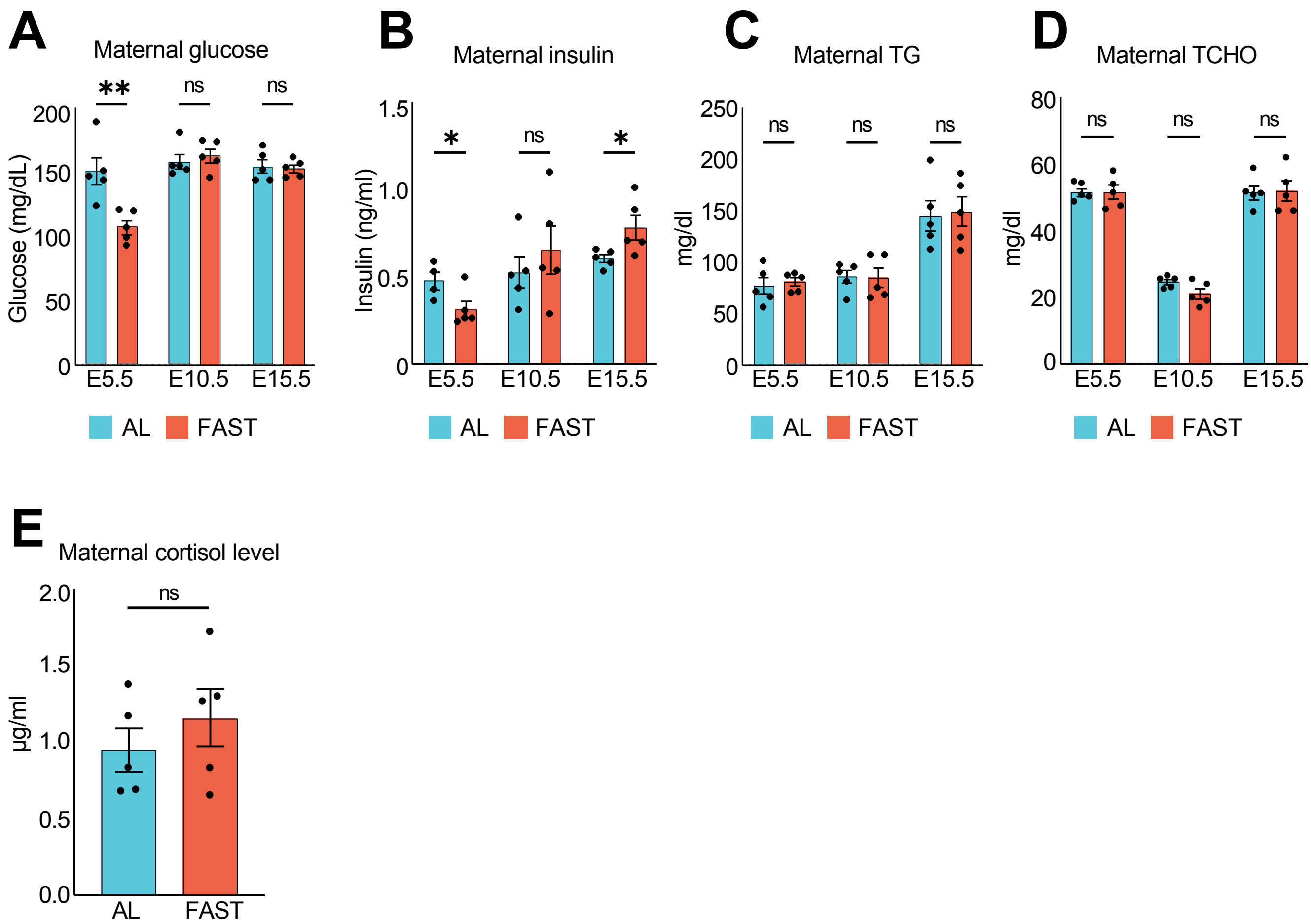
A
Maternal glucose
Glucose (mg/dL)
**
ns
ns
0
50
100
150
200
E5.5
E10.5
E15.5
AL
FAST
B
Maternal insulin
Insulin (ng/ml)
*
ns
*
0
0.5
1.0
1.5
E5.5
E10.5
E15.5
AL
FAST
C
Maternal TG
mg/dl
ns
ns
ns
0
50
100
150
200
250
E5.5
E10.5
E15.5
AL
FAST
D
Maternal TCHO
mg/dl
ns
ns
ns
0
20
40
60
80
E5.5
E10.5
E15.5
AL
FAST
E
Maternal cortisol level
μg/ml
ns
0.0
0.5
1.0
1.5
2.0
AL
FAST

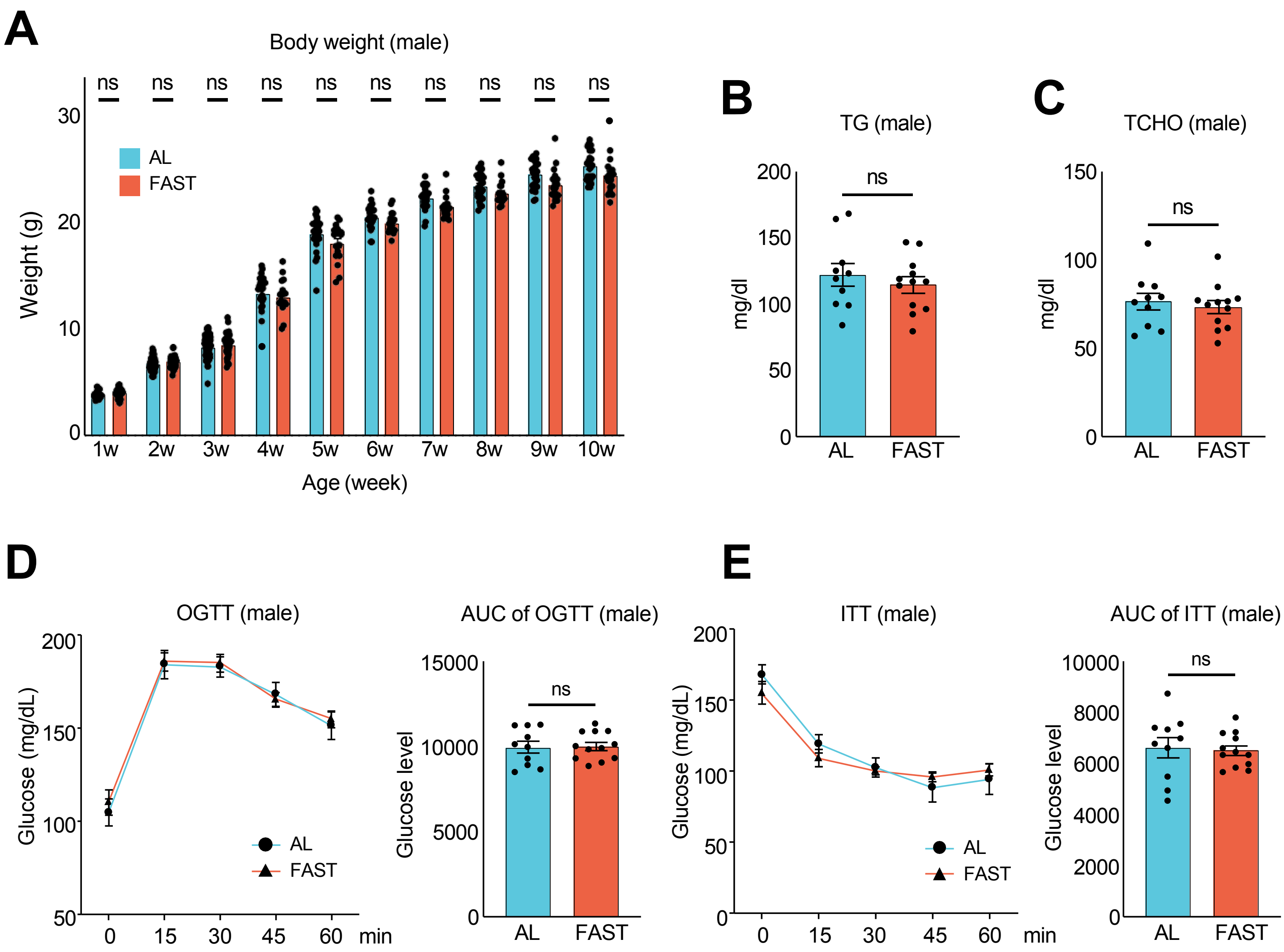
A
Body weight (male)
ns ns ns ns ns ns ns ns ns ns
AL
FAST
Weight (g)
0
10
20
30
1w 2w 3w 4w 5w 6w 7w 8w 9w 10w
Age (week)
B
TG (male)
mg/dl
0
50
100
150
200
ns
AL FAST
C
TCHO (male)
mg/dl
0
50
100
150
ns
AL FAST
D
OGTT (male)
Glucose (mg/dL)
50
100
150
200
0 15 30 45 60 min
AL
FAST
AUC of OGTT (male)
Glucose level
0
5000
10000
15000
ns
AL FAST
E
ITT (male)
Glucose (mg/dL)
0
50
100
150
200
0 15 30 45 60 min
AL
FAST
AUC of ITT (male)
Glucose level
0
2000
4000
6000
8000
10000
ns
AL FAST

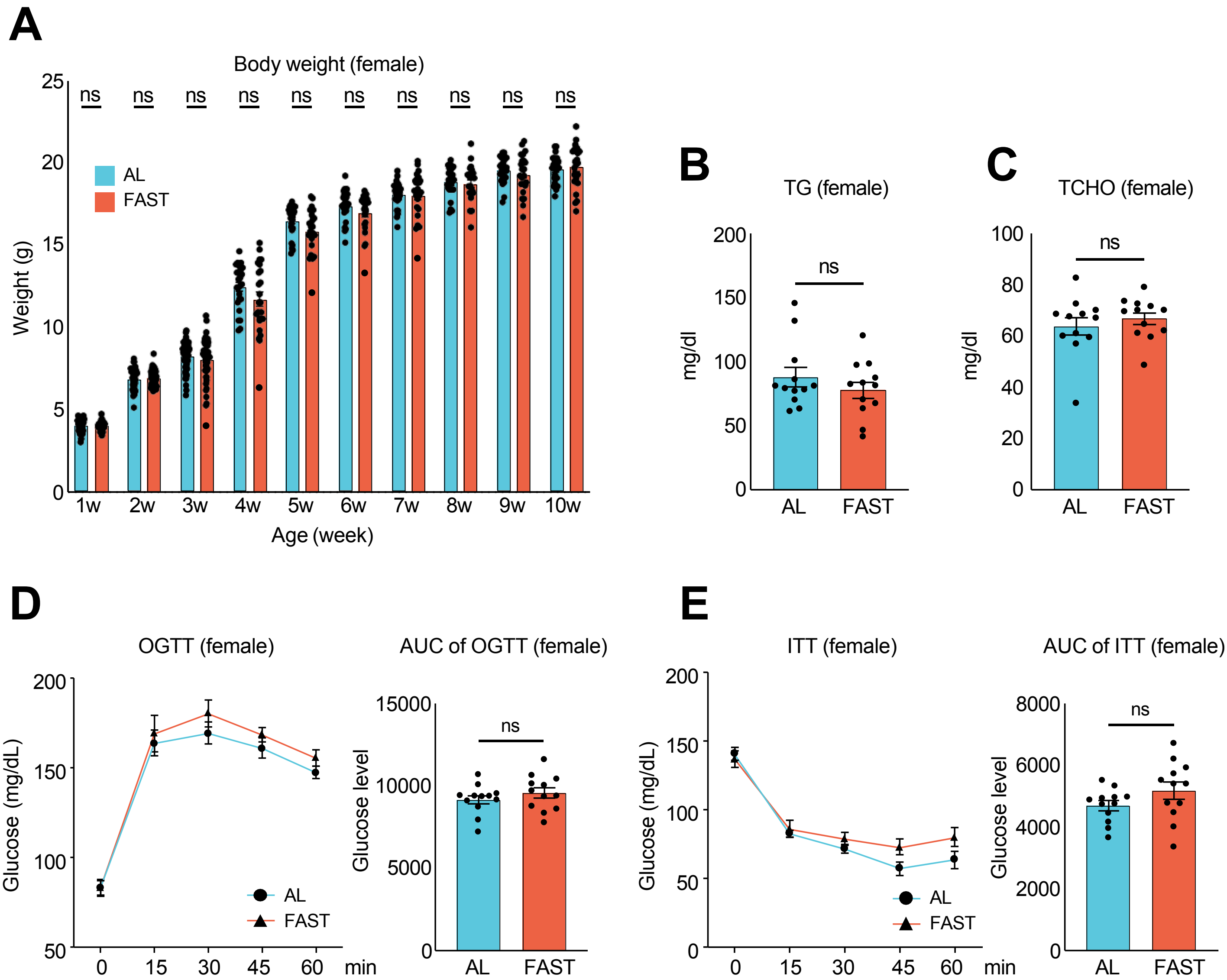
A
Body weight (female)
ns ns ns ns ns ns ns ns ns ns
AL
FAST
Weight (g)
0
5
10
15
20
25
1w 2w 3w 4w 5w 6w 7w 8w 9w 10w
Age (week)
B
TG (female)
mg/dl
0
50
100
150
200
ns
AL FAST
C
TCHO (female)
mg/dl
0
20
40
60
80
100
ns
AL FAST
D
OGTT (female)
Glucose (mg/dL)
50
100
150
200
0 15 30 45 60 min
AL
FAST
AUC of OGTT (female)
Glucose level
0
5000
10000
15000
ns
AL FAST
E
ITT (female)
Glucose (mg/dL)
0
50
100
150
200
0 15 30 45 60 min
AL
FAST
AUC of ITT (female)
Glucose level
0
2000
4000
6000
8000
ns
AL FAST

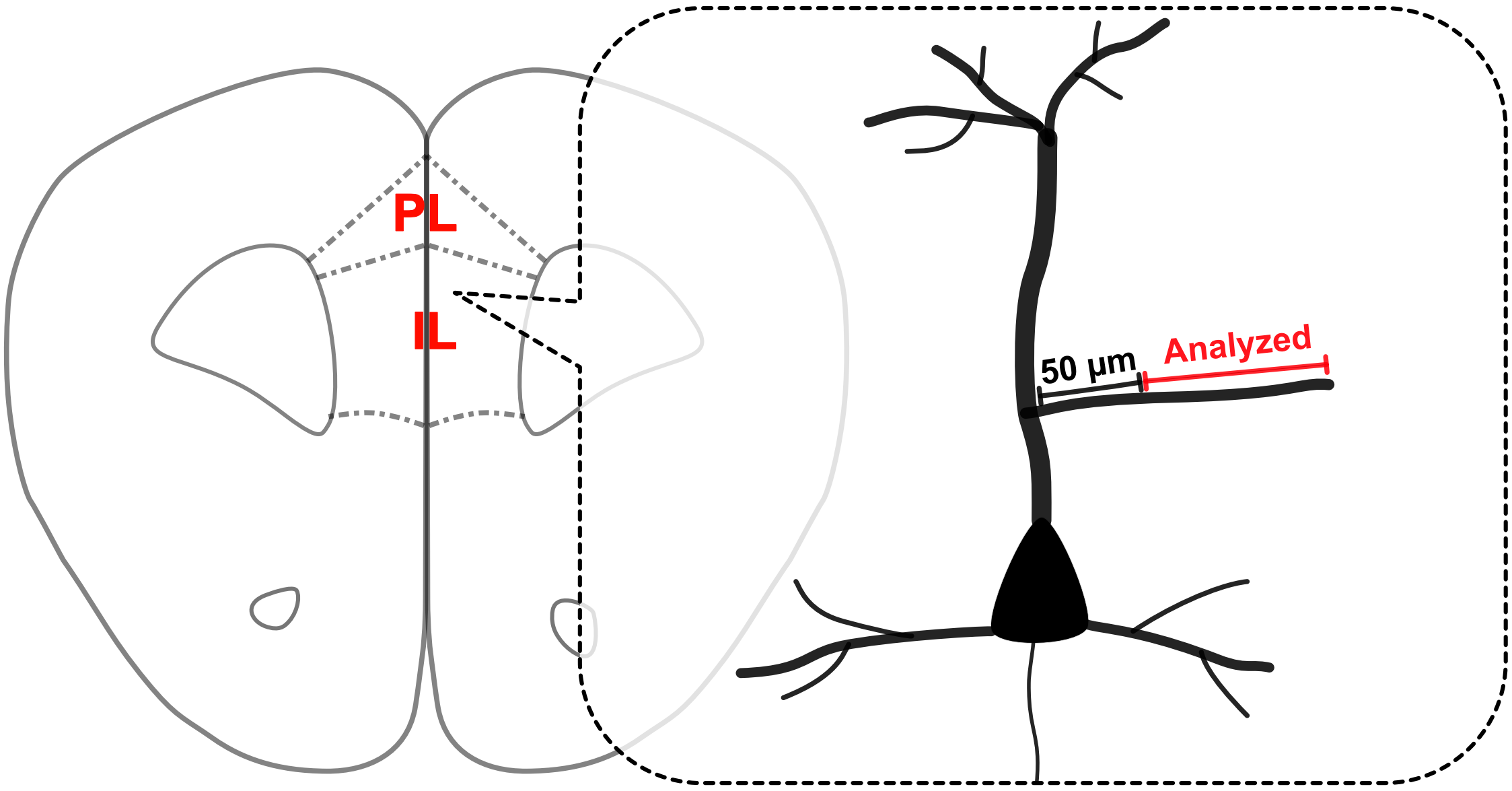
PL
IL
50 µm
Analyzed

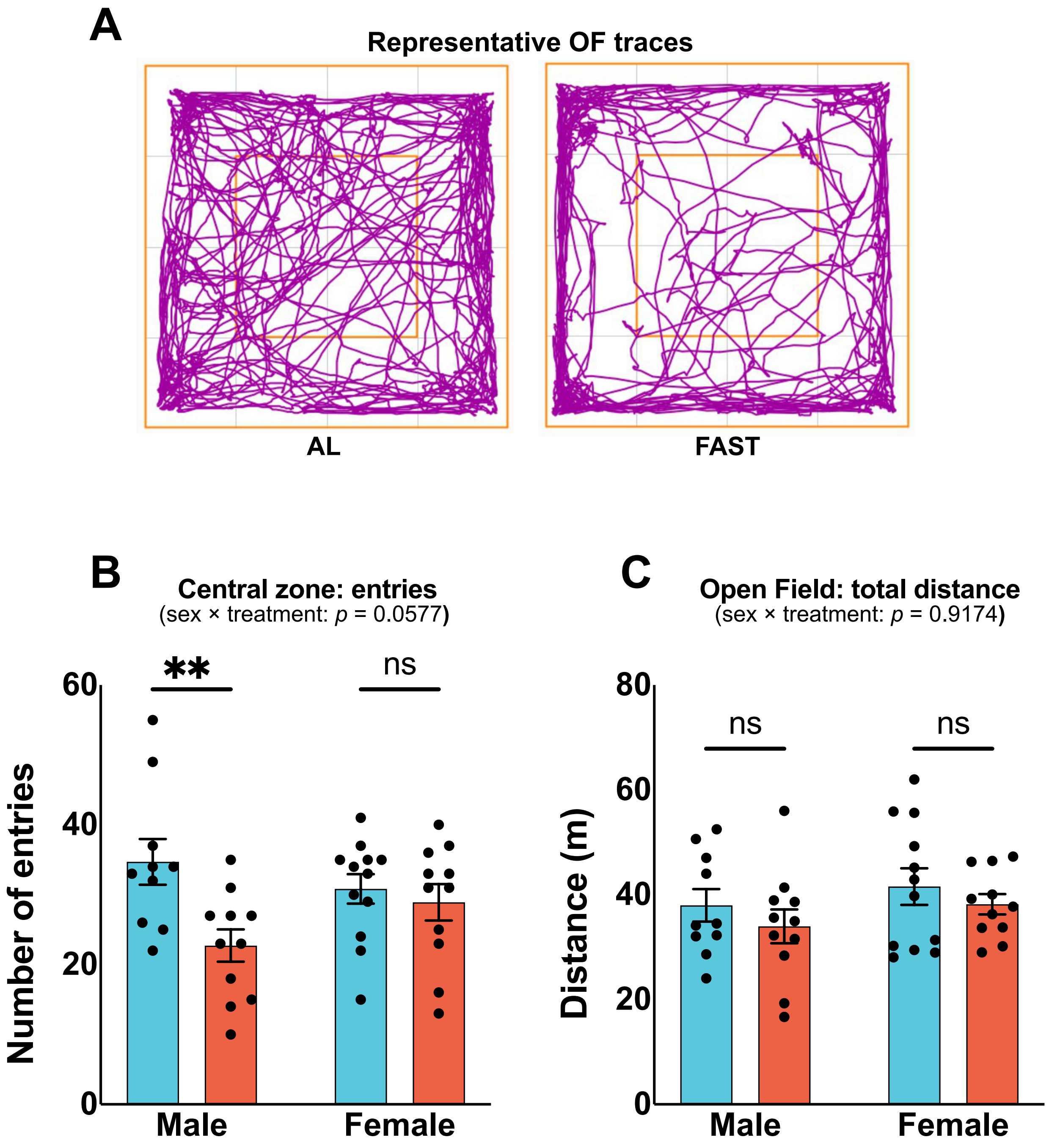
A
Representative OF traces
AL
FAST
B
Central zone: entries
(sex × treatment: p = 0.0577)
**
ns
60
40
20
0
Number of entries
Male
Female
C
Open Field: total distance
(sex × treatment: p = 0.9174)
ns
ns
80
60
40
20
0
Distance (m)
Male
Female

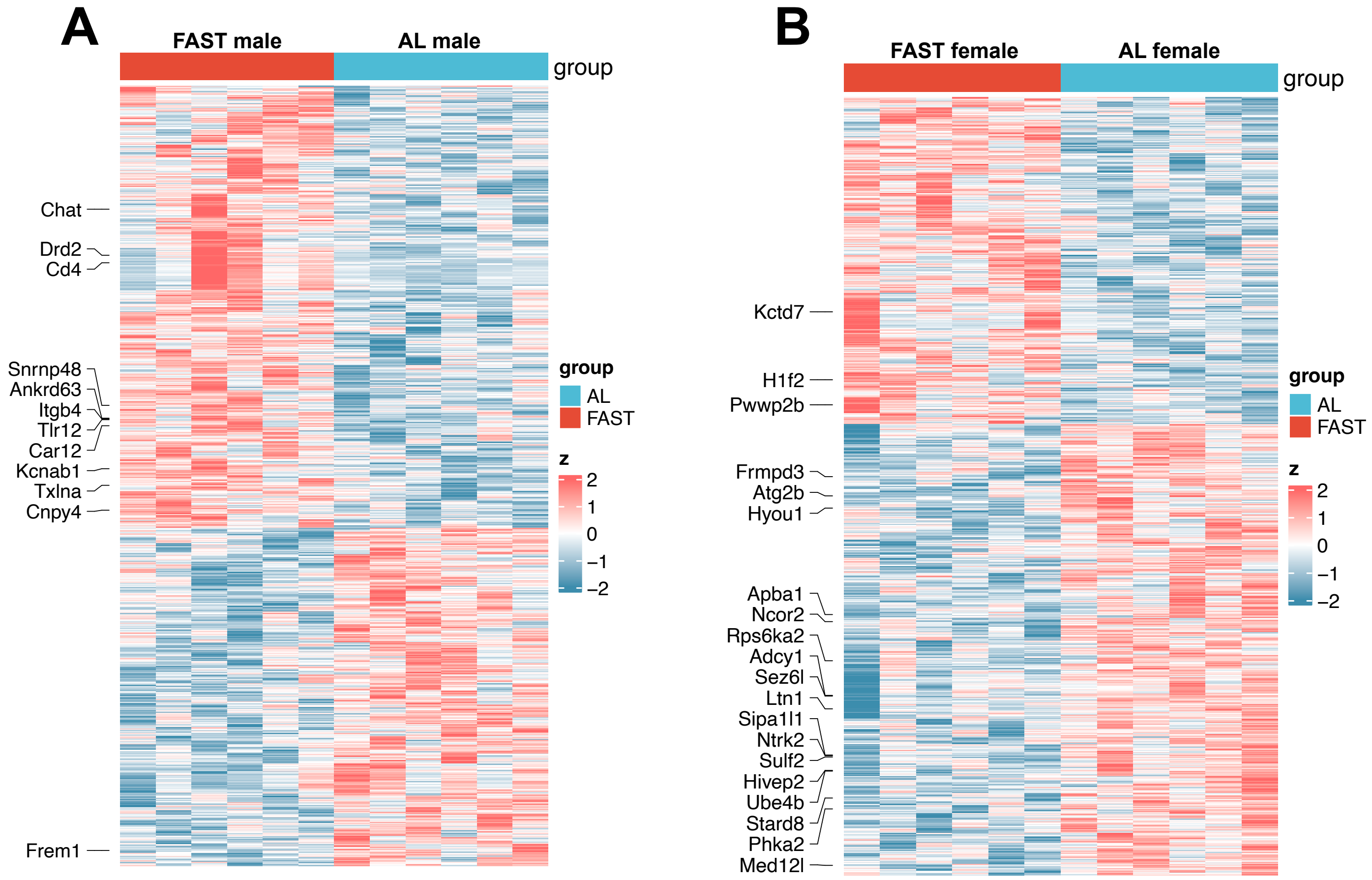
A
FAST male
AL male
group
Chat
Drd2
Cd4
Snrnp48
Ankrd63
Itgb4
Tlr12
Car12
Kcnab1
Txlna
Cnpy4
Frem1
group
AL
FAST
z
2
1
0
−1
−2
B
FAST female
AL female
group
Kctd7
H1f2
Pwwp2b
Frmpd3
Atg2b
Hyou1
Apba1
Ncor2
Rps6ka2
Adcy1
Sez6l
Ltn1
Sipa1l1
Ntrk2
Sulf2
Hivep2
Ube4b
Stard8
Phka2
Med12l
group
AL
FAST
z
2
1
0
−1
−2

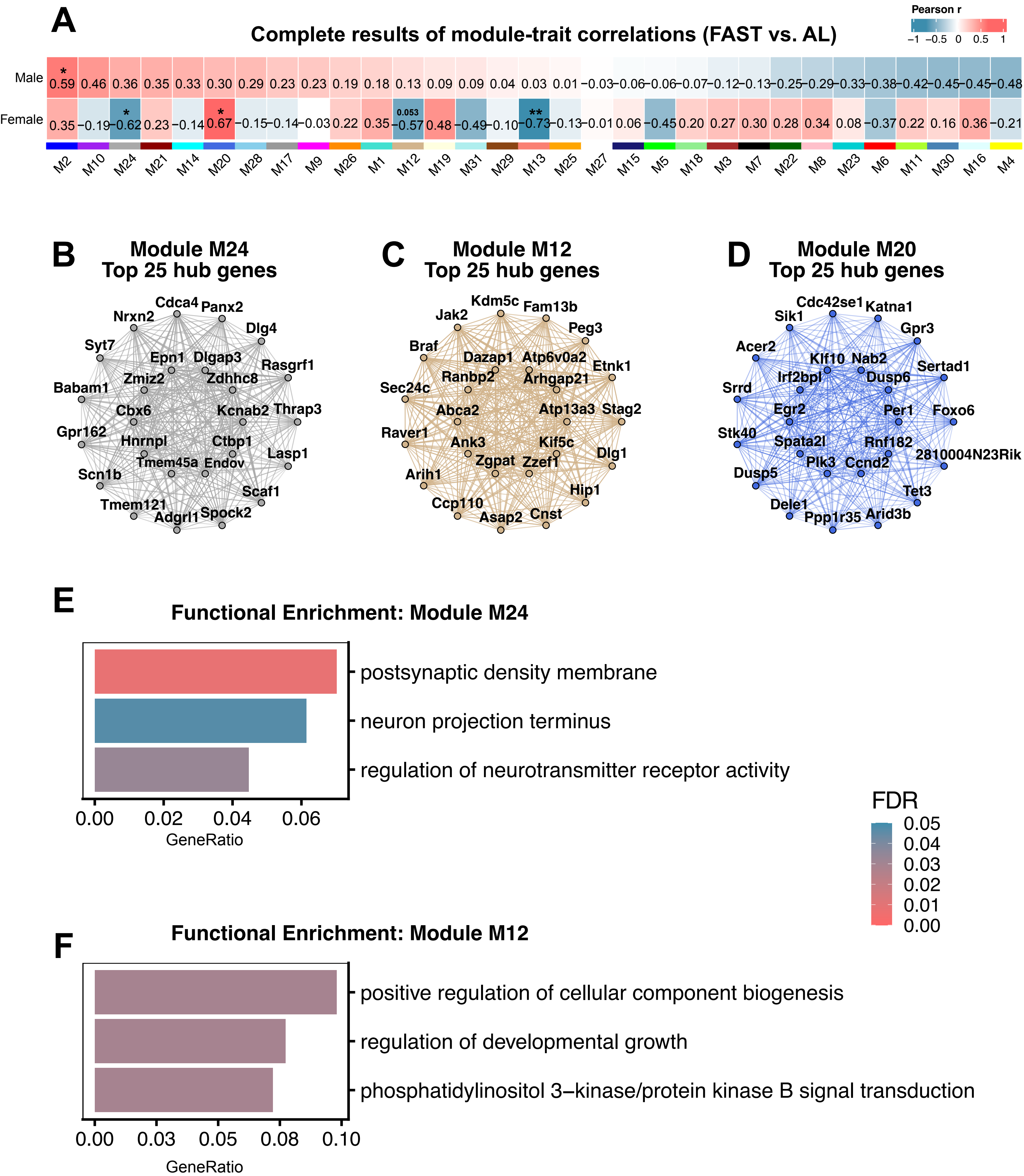

A
Complete results of module-trait correlations (FAST vs. AL)
Pearson r
−1 −0.5 0 0.5 1
Male
* 0.59 0.46 0.36 0.35 0.33 0.30 0.29 0.23 0.23 0.19 0.18 0.13 0.09 0.09 0.04 0.03 0.01 −0.03 −0.06 −0.06 −0.07 −0.12 −0.13 −0.25 −0.29 −0.33 −0.38 −0.42 −0.45 −0.45 −0.48
Female
0.35 −0.19 * −0.62 0.23 −0.14 * 0.67 −0.15 −0.14 −0.03 0.22 0.35 0.053 −0.57 0.48 −0.49 −0.10 ** −0.73 −0.13 −0.01 0.06 −0.45 0.20 0.27 0.30 0.28 0.34 0.08 −0.37 0.22 0.16 0.36 −0.21
M2 M10 M24 M21 M14 M20 M28 M17 M9 M26 M1 M12 M19 M31 M29 M13 M25 M27 M15 M5 M18 M3 M7 M22 M8 M23 M6 M11 M30 M16 M4
B
Module M24
Top 25 hub genes
Cdca4 Panx2 Nrxn2 Dlg4 Syt7 Epn1 Dlgap3 Rasgrf1 Zmiz2 Zdhhc8 Babam1 Cbx6 Kcnab2 Thrap3 Gpr162 Hnrnpl Ctbp1 Lasp1 Tmem45a Endov Scn1b Scaf1 Tmem121 Adgrl1 Spock2
C
Module M12
Top 25 hub genes
Kdm5c Fam13b Jak2 Peg3 Braf Dazap1 Atp6v0a2 Etnk1 Ranbp2 Arhgap21 Sec24c Abca2 Atp13a3 Stag2 Raver1 Ank3 Kif5c Dlg1 Zgpat Zzef1 Arih1 Hip1 Ccp110 Asap2 Cnst
D
Module M20
Top 25 hub genes
Cdc42se1 Katna1 Sik1 Gpr3 Acer2 Klf10 Nab2 Sertad1 Irf2bpl Dusp6 Srrd Egr2 Per1 Foxo6 Stk40 Spata2l Rnf182 2810004N23Rik Plk3 Ccnd2 Dusp5 Tet3 Dele1 Ppp1r35 Arid3b
E
Functional Enrichment: Module M24
postsynaptic density membrane
neuron projection terminus
regulation of neurotransmitter receptor activity
0.00 0.02 0.04 0.06
GeneRatio
FDR
0.05
0.04
0.03
0.02
0.01
0.00
F
Functional Enrichment: Module M12
positive regulation of cellular component biogenesis
regulation of developmental growth
phosphatidylinositol 3−kinase/protein kinase B signal transduction
0.00 0.03 0.05 0.08 0.10
GeneRatio

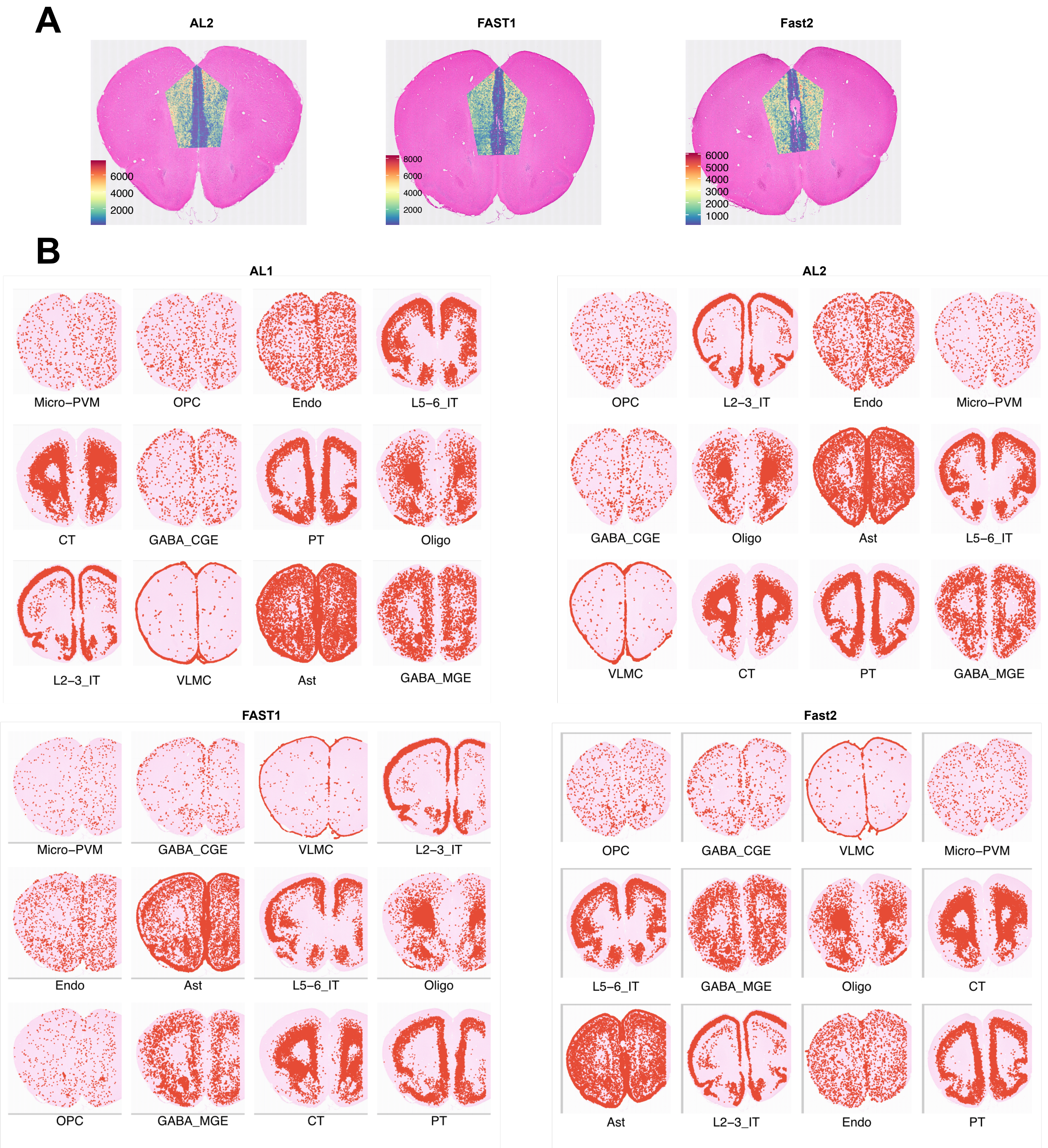
A
AL2
FAST1
Fast2
6000
4000
2000
8000
6000
4000
2000
6000
5000
4000
3000
2000
1000
B
AL1
Micro−PVM
OPC
Endo
L5−6_IT
CT
GABA_CGE
PT
Oligo
L2−3_IT
VLMC
Ast
GABA_MGE
AL2
OPC
L2−3_IT
Endo
Micro−PVM
GABA_CGE
Oligo
Ast
L5−6_IT
VLMC
CT
PT
GABA_MGE
FAST1
Micro−PVM
GABA_CGE
VLMC
L2−3_IT
Endo
Ast
L5−6_IT
Oligo
OPC
GABA_MGE
CT
PT
Fast2
OPC
GABA_CGE
VLMC
Micro−PVM
L5−6_IT
GABA_MGE
Oligo
CT
Ast
L2−3_IT
Endo
PT

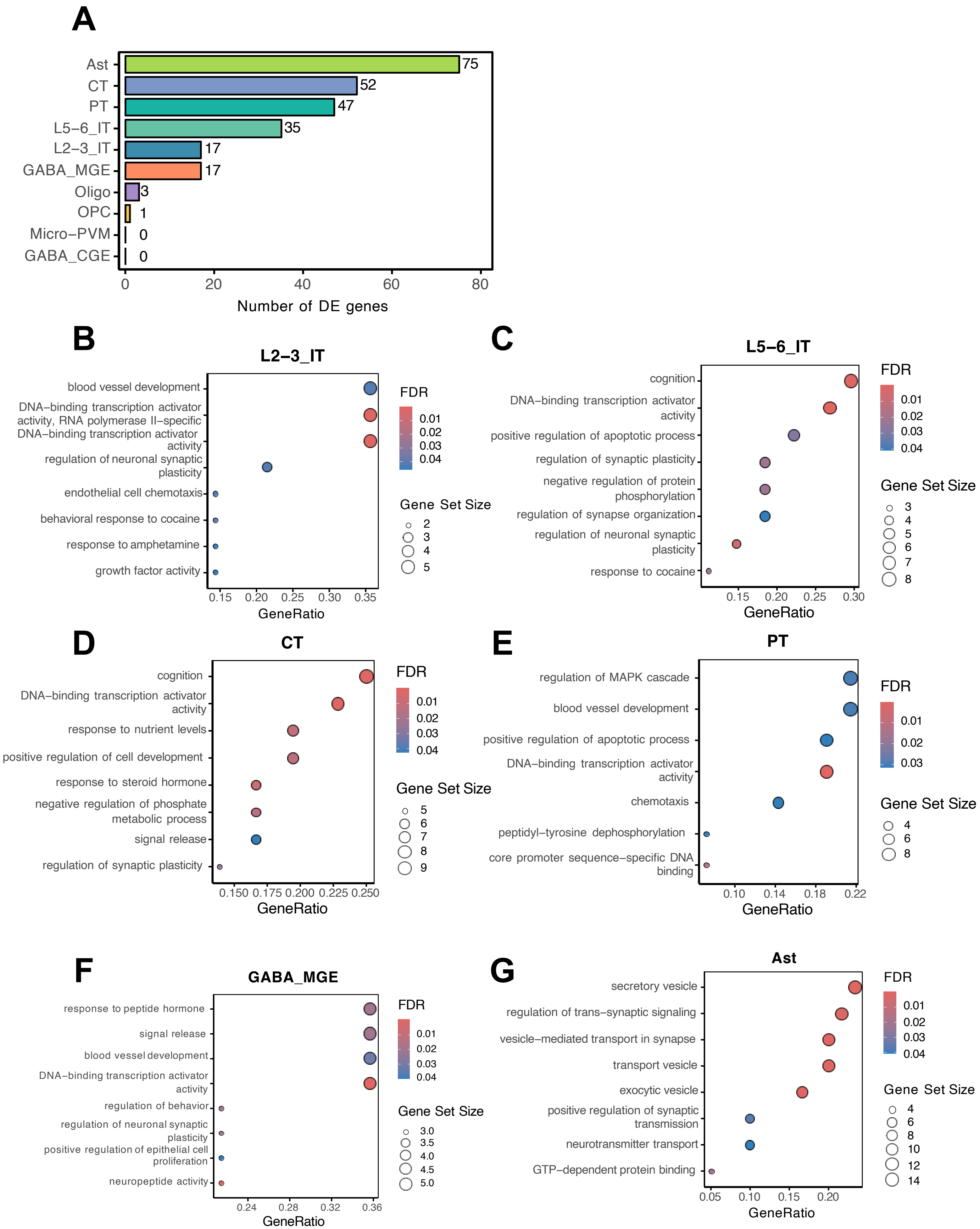
A
Ast
CT
PT
L5−6_IT
L2−3_IT
GABA_MGE
Oligo
OPC
Micro−PVM
GABA_CGE
75
52
47
35
17
17
3
1
0
0
0
20
40
60
80
Number of DE genes
B
L2−3_IT
blood vessel development
DNA−binding transcription activator activity, RNA polymerase II−specific
DNA−binding transcription activator activity
regulation of neuronal synaptic plasticity
endothelial cell chemotaxis
behavioral response to cocaine
response to amphetamine
growth factor activity
0.15 0.20 0.25 0.30 0.35
GeneRatio
FDR
0.01
0.02
0.03
0.04
Gene Set Size
2
3
4
5
C
L5−6_IT
cognition
DNA−binding transcription activator activity
positive regulation of apoptotic process
regulation of synaptic plasticity
negative regulation of protein phosphorylation
regulation of synapse organization
regulation of neuronal synaptic plasticity
response to cocaine
0.15 0.20 0.25 0.30
GeneRatio
FDR
0.01
0.02
0.03
0.04
Gene Set Size
3
4
5
6
7
8
D
CT
cognition
DNA−binding transcription activator activity
response to nutrient levels
positive regulation of cell development
response to steroid hormone
negative regulation of phosphate metabolic process
signal release
regulation of synaptic plasticity
0.150 0.175 0.200 0.225 0.250
GeneRatio
FDR
0.01
0.02
0.03
0.04
Gene Set Size
5
6
7
8
9
E
PT
regulation of MAPK cascade
blood vessel development
positive regulation of apoptotic process
DNA−binding transcription activator activity
chemotaxis
peptidyl−tyrosine dephosphorylation
core promoter sequence−specific DNA binding
0.10 0.14 0.18 0.22
GeneRatio
FDR
0.01
0.02
0.03
Gene Set Size
4
6
8
F
GABA_MGE
response to peptide hormone
signal release
blood vessel development
DNA−binding transcription activator activity
regulation of behavior
regulation of neuronal synaptic plasticity
positive regulation of epithelial cell proliferation
neuropeptide activity
0.24 0.28 0.32 0.36
GeneRatio
FDR
0.01
0.02
0.03
0.04
Gene Set Size
3.0
3.5
4.0
4.5
5.0
G
Ast
secretory vesicle
regulation of trans−synaptic signaling
vesicle−mediated transport in synapse
transport vesicle
exocytic vesicle
positive regulation of synaptic transmission
neurotransmitter transport
GTP−dependent protein binding
0.05 0.10 0.15 0.20
GeneRatio
FDR
0.01
0.02
0.03
0.04
Gene Set Size
4
6
8
10
12
14

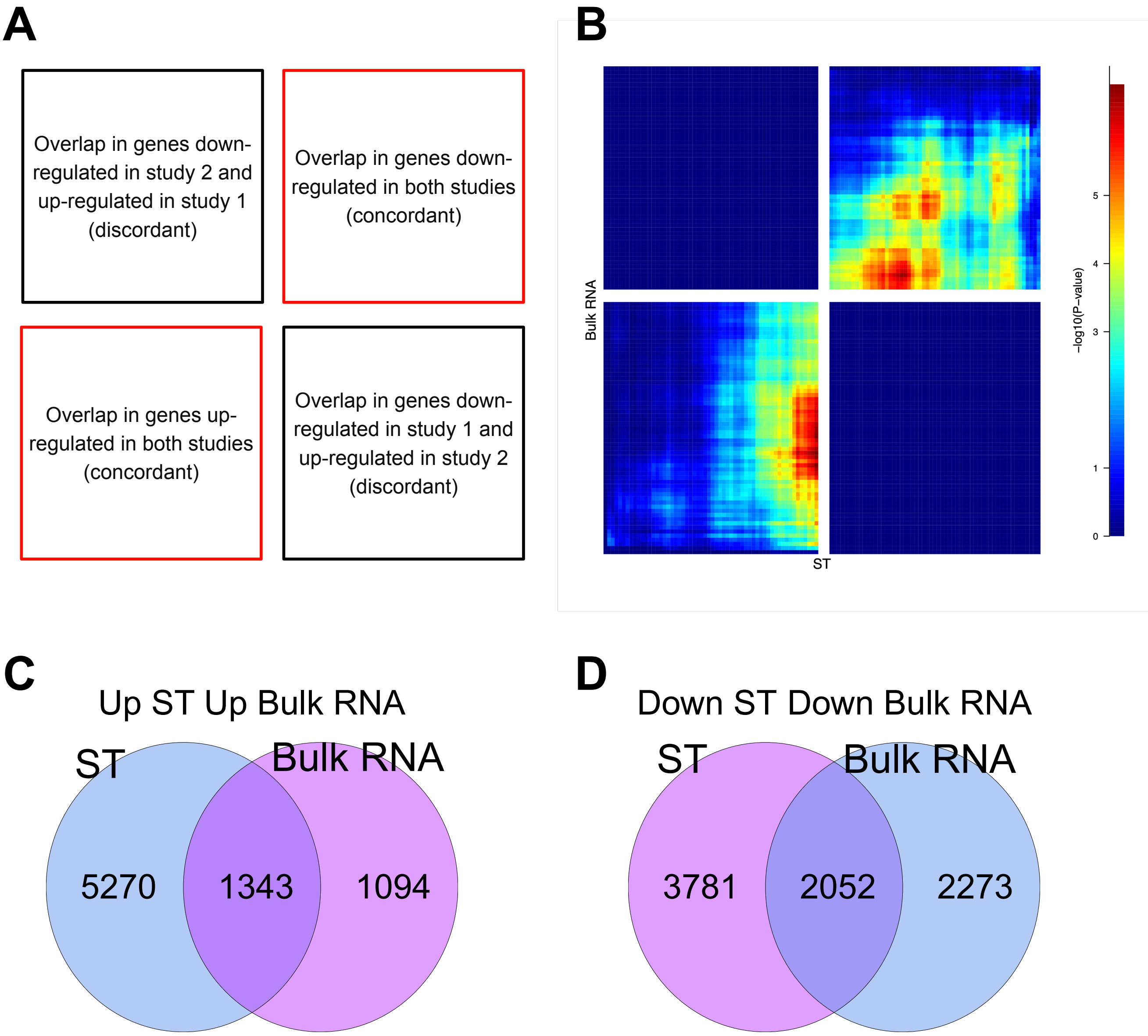

A
Overlap in genes down-regulated in study 2 and up-regulated in study 1 (discordant)
Overlap in genes down-regulated in both studies (concordant)
Overlap in genes up-regulated in both studies (concordant)
Overlap in genes down-regulated in study 1 and up-regulated in study 2 (discordant)
B
Bulk RNA
ST
−log10(P−value)
0
1
3
4
5
C
Up ST Up Bulk RNA
ST
Bulk RNA
5270
1343
1094
D
Down ST Down Bulk RNA
ST
Bulk RNA
3781
2052
2273

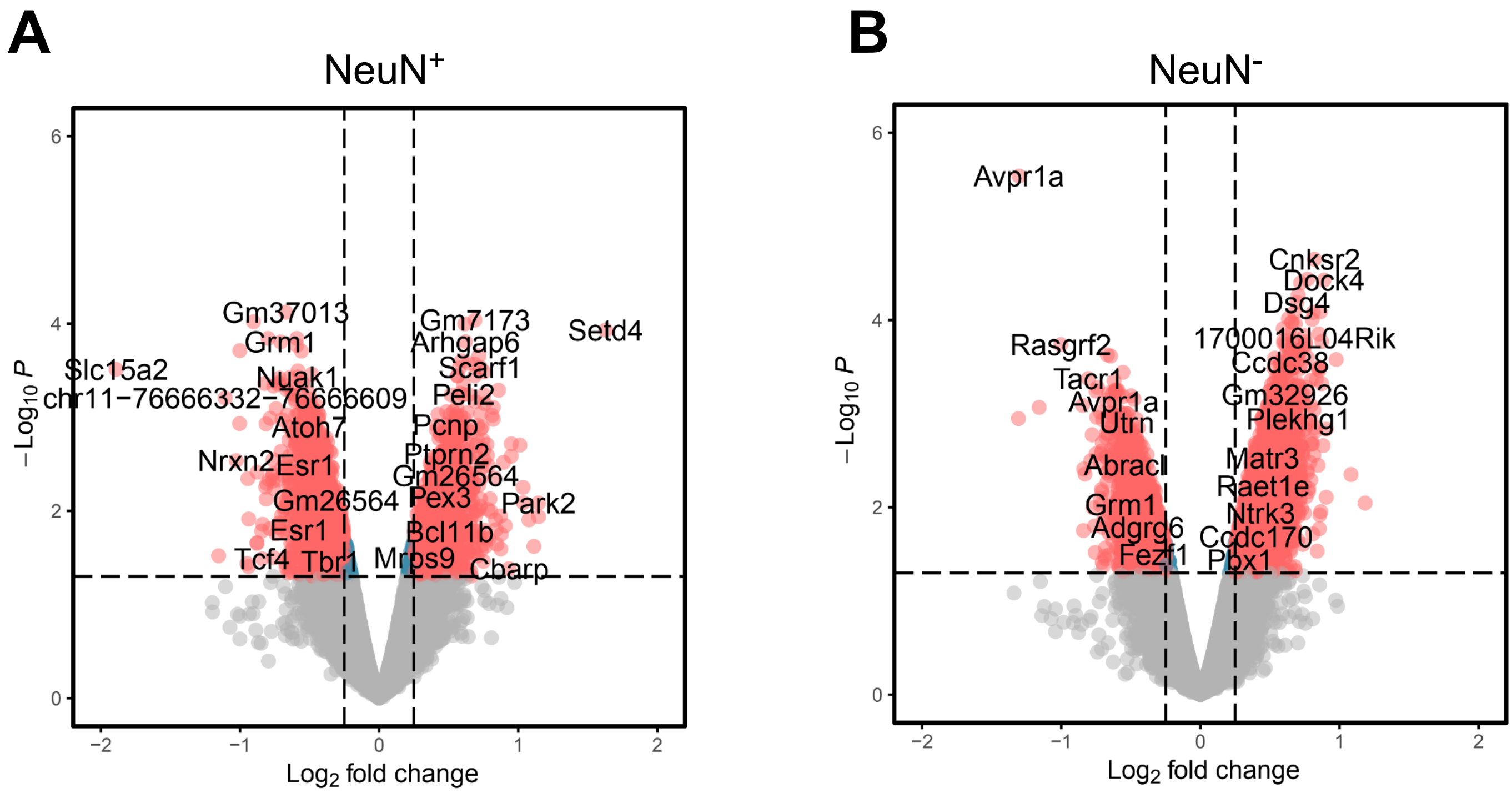

A
NeuN+
B
NeuN-
Log2 fold change
−Log10 P
Log2 fold change
−Log10 P
